# Pecan secondary metabolites influenced the population of *Zeuzera coffeae* by affecting the structure and function of the larval gut microbiota

**DOI:** 10.3389/fmicb.2024.1379488

**Published:** 2024-04-12

**Authors:** Jie Wang, Shouke Zhang, Junqia Kong, Jun Chang

**Affiliations:** ^1^State Key Laboratory of Subtropical Silviculture, Zhejiang A&F University, Hangzhou, China; ^2^College of Landscape Architecture, Zhejiang A&F University, Hangzhou, China; ^3^Research Institute of Subtropical Forestry, Chinese Academy of Forestry, Hangzhou, China

**Keywords:** *Zeuzera coffeae*, *Carya illinoinensis*, plant secondary metabolites, gut microbiota, resistant cultivars

## Abstract

**Background:**

The plant secondary metabolites (PSMs), as important plant resistance indicators, are important targets for screening plant insect resistance breeding. In this study, we aimed to investigate whether the population of *Zeuzera coffeae* (ZC) is affected by different varieties of *Carya illinoinensis* PSMs content. At the same time, the structure and function of the gut microbiome of ZC were also analyzed in relation to different pecan varieties.

**Methods:**

We counted the populations of ZC larvae in four pecan varieties and determined the content of four types of PSMs. The structure and function of the larval gut microbiota were studied in connection to the number of larvae and the content of PSMs. The relationships were investigated between larval number, larval gut microbiota, and PSM content.

**Results:**

We found that the tannins, total phenolics, and total saponins of 4 various pecans PSMs stifled the development of the ZC larval population. The PSMs can significantly affect the diversity and abundance of the larval gut microbiota. Enrichment of ASV46 (*Pararhizobium* sp.), ASV994 (*Olivibacter* sp.), ASV743 (*Rhizobium* sp.), ASV709 (*Rhizobium* sp.), ASV671 (*Luteolibacter* sp.), ASV599 (*Agrobacterium* sp.), ASV575 (*Microbacterium* sp.), and ASV27 (*Rhizobium* sp.) in the gut of larvae fed on high-resistance cultivars was positively associated with their tannin, total saponin, and total phenolic content. The results of the gut microbiome functional prediction for larvae fed highly resistant pecan varieties showed that the enriched pathways in the gut were related to the breakdown of hazardous chemicals.

**Conclusion:**

Our findings provide further evidence that pecan PSMs influence the structure and function of the gut microbiota, which in turn affects the population stability of ZC. The study’s findings can serve as a theoretical foundation for further work on selecting ZC-resistant cultivars and developing green management technology for ZC.

## 1 Introduction

Growth and development of herbivorous insects are indirectly affected by the composition of their gut microbiota, which plays an essential role in the metabolism of nutrition, the breakdown of toxins, and the spread of pesticide resistance (Shin et al., 2011; Thong-On et al., 2012; Schuman and Baldwin, 2016; Bai et al., 2020). In recent years, many studies have focused on the adaptation of gut microbiota to plant secondary metabolites (PSMs) in coordination with phytophagous insects (Zhang et al., 2023). However, the study on the effect of PSMs on gut microbiota of pests and thus inhibiting the normal growth of pests was neglected (Schuman and Baldwin, 2016; Mason et al., 2019; Mason, 2020). With the development of molecular breeding, it is possible to regulate the accumulation of some PSMs (Ahmar et al., 2020; Li et al., 2020). Through the effective accumulation of insect resistant substances, the gut microbiota of pests can be interfered with, and then the nutrient accumulation of pests can be affected, which may be an effective means to effectively prevent plant-eating pests, especially the trunk-boring pests which are difficult to control (Singh and Kaur, 2018; Kortbeek et al., 2021).

When phytophagous insects feed on plants or lay eggs, they trigger the activation of the induced resistance mechanism, resulting in a stress defense response (Zhang and Liu, 2006; Bonaventure, 2012). This response produces and releases various PSMs, such as indole, methylsalicylic acid, leaf alcohol, trans-3-hexenol, monophosphores, and sesquiterpenes, that have evocative effects on the feeding and laying choices of insects (Lee et al., 2015; Beran et al., 2019; Yang et al., 2020; Mollah et al., 2021). However, several studies have demonstrated that phytophagous insects possess a robust capacity for learning and adaptability (Tokuda et al., 2022). Moreover, after multiple generations of adaptation to suitable hosts, such hosts may lose their initial resistance, particularly in the case of responses induced by certain plants (Despres et al., 2007). Consequently, stress resistance indices represent the primary means of assessing participation in resistant variety selection (Zhang H. et al., 2020; Zhang S. et al., 2020).

Pecan, as an important economic crop, is rich in nutrients and has high value (Ortiz-Quezada et al., 2011; Huang et al., 2019). To increase the economic returns of forestry, China introduced pecan in Jiangxi in 1890 and began large-scale cultivation in 1996 (Zhang et al., 2003). With the increase in cultivation area, the stem-boring pest *Zeuzera coffeae* (ZC) has gradually become a major pest affecting the healthy development of the pecan industry (Ju et al., 2019). Currently, chemical control of stem-boring pests has shown limited effectiveness and is not conducive to the safety of forest ecosystems and the green, pollution-free production of pecan products (Rebek et al., 2012). Therefore, the selection of a set of effective pest resistance indicators is particularly important. The concentrations of PSMs, including tannins, phenolics, and flavonoids, vary among different strains of pecans, as is widely recognized (Li et al., 2022). Investigating whether the population of ZC can be impacted by PSMs of various types of pecans through their influence on the gut microbiota of ZC larvae is crucial, especially for assessing the effectiveness of insect resistance traits in pecans.

Over a period of 5 years, in this study, we observed changes in ZC population on various pecan cultivars. We evaluated the levels of four PSMs, namely tannins, total phenols, total saponins, and cellulose, which are known for their insect resistance properties (Yonekura-Sakakibara et al., 2019; Li et al., 2022; Phukan et al., 2023). Furthermore, we analyzed the connections between these parameters and the gut microbiota structure and function of ZC. The study’s development may determine whether the PSM content in various pecan varieties impacts the gut microbiota structure of ZC, thereby strengthening the theory of using PSMs as a measure for insect resistance breeding in pecans.

## 2 Materials and methods

### 2.1 Sample collection

Surveys were conducted on 4 pecan varieties, Kanza (KZ), Number 27 (EQ), Pawnee (BN), and Mahan (MH), in plantations located at 2 sites (117°23′37.12″, 32°11′50.38″; 118°7′43.08″, 32°3′5.60″) in Anhui, China, and one site (119°32′32.93″, 29°5′21.04″) in Zhejiang, China. The pecan forests were essentially in a natural state without the application of pesticides for disease, insect, or weed control. During the time when ZC larvae posed a threat to pecan, branches with larval infestation were randomly selected from the three study sites as experimental samples. After bringing the samples back to the laboratory, the branches were dissected under sterile conditions, and the larvae were taken out for test. The larvae gut was dissected in PBS buffer, labeled, and stored at −80°C for test (Zhang et al., 2021). The branches of the 4 varieties of pecan were classified and labeled for the preparation of PSMs content analysis.

### 2.2 Extraction and analysis of ZC gut microbiota

To obtain all the DNA from the ZC larvae gut microbiota, we followed the extraction protocol of the QIAGEN DNeasy Blood & Tissue Kit as a reference. We dissected the gut in PBS buffer and collected it in sterile PE tubes with 1 mL PBS. Each tube contained the gut of 5 larvae guts to ensure DNA quality. We homogenized the collected material and performed centrifugation at 7,500 rpm at 4°C. Finally, we transferred the supernatant into new sterile PE tubes to extract the total DNA. Afterward, the purity and concentration of the DNA samples were evaluated. The Pacbio Sequel II platform was used for sequencing, with CLR (continuous long read) mode. PCR amplification of the nearly full-length bacterial 16S rRNA genes was performed using the forward primer 27F (5′-AGAGTTTGATCMTGGCTCAG-3′) and the reverse primer 1492R (5′-ACCTTGTTACGACTT-3′). The extracted DNA was amplified with a two-step PCR, incorporating sample-specific 16-bp barcodes into the forward and reverse primers for multiplex sequencing in the second PCR step. Both steps of the PCR contained 5 μL of Q5 reaction buffer (5×), 5 μL of Q5 High-Fidelity GC buffer (5×), 0.25 μL of Q5 High-Fidelity DNA Polymerase (5 U/μl), 2 μL (2.5 mM) of dNTPs, 1 μL (10 uM) of each Forward and Reverse primer, 2 μL of DNA Template, and 8.75 μL of ddH2O. Thermal cycling involved an initial denaturation at 98°C for 2 min, followed by 25/10 cycles (for the first and second amplification steps, respectively). Each cycle consisted of denaturation at 98°C for 30 s, annealing at 55°C for 30 s, and extension at 72°C for 90 s. The process concluded with a final extension of 5 min at 72°C. The PCR amplicons were purified using Agencourt AMPure Beads (Beckman Coulter, Indianapolis, IN) and quantified using the PicoGreen dsDNA Assay Kit (Invitrogen, Carlsbad, CA, United States). Following the individual quantification step, the amplicons were combined in equal proportions. The Single Molecule Real Time (SMRT) sequencing technology was then utilized on the PacBio Sequel platform at Shanghai Personal Biotechnology Co., Ltd. (Shanghai, China). To reduce the sequencing error rate, PacBio circular consensus sequencing (CCS) reads were obtained from the multiple alignments of sub-reads. In CCS, the DNA polymerase reads a ligated circular DNA template multiple times, generating a consensus sequence from multiple reads of a single molecule. Raw sequences were processed through the PacBio SMRT Link portal (version 5.0.1.9585), filtered for a minimum of 3 passes, and a minimum predicted accuracy of 99%. The predicted accuracy of 99% is the threshold below which a CCS is considered noise. The PacBio platform generated files, which were then used to trim amplicon sizes by removing sequences longer than 2,000 bp. The sequencing results were deposited in Bio Project accession numbers (PRJNA1027071) of the NCBI Web database.

### 2.3 Determination of PSMs content in injured pecan branches

To determine the amount of plant tannin present, we weighed 0.1 g of tissue and add it to 1 mL of distilled water. Mix thoroughly and extract it in a water bath at 80°C, for 30 min. Centrifuge the solution at 10,000 rpm and 25°C, for 10 min. Collect the resulting supernatant and analyze it to extract the tannin content, using the Tannin content Test kit (Suzhou Michy Biomedical Technology Co., Ltd., Suzhou, China). Tannins mix with molybdic acid in a basic surrounding to make a blue compound that has the highest absorption at 765 nm (Arbenz and Avérous, 2015; Huang et al., 2022). To figure out the amount of tannin in the sample, we measured the absorbance value at 765 nm.

For determining the total phenol content in plants, we dried the sample, crushed it, and filtered it through a 40-mesh screen. We took around 0.02 g of the sample and added it to 1 mL of extraction solution. After spinning at 10,000 g at 25°C for 10 min, we collected the liquid that rose to the top for testing. We used Plant Total Phenols content test kit (Suzhou Michy Biomedical Technology Co., Ltd., Suzhou, China) to extract the full number of phenols in the samples. Phenols react with ammonium molybdate in alkaline conditions to produce a blue substance that peaks at an absorption level of 765 nm. To find out the total phenol content in the sample, we measured the absorbance value at 765 nm (Ning et al., 2023).

To find out how much cellulose a plant contains, weigh around 0.1 g of the sample. Use the cellulose content test kit made by Cellulose content test kit (Suzhou Michy Biomedical Technology Co., Ltd., Suzhou, China) to extract cellulose content. Cellulose is a type of sugar with β-glucose parts, which can be broken down into β-glucose under acid and heat. β-glucose turns into β-hydroxymethylfurfural compounds with strong acid conditions when it becomes dehydrated. The β-hydroxymethylfurfural compounds react with anthrone to create derivatives that are measured to determine the cellulose content (Mishra et al., 2019).

To determine the total saponin content of the plant, the samples should be firstly dried, grinded, and sieved. Then, add roughly 0.05 g of the sample to 1 mL of extraction solution and use ultrasonic extraction for 1 h. After spinning at 10,000 rpm for 10 min at 25°C, the top liquid layer was gathered for analysis. Saponin quantity was extracted with Total saponins content test kit saponin test kit. Ultrasonic technique was utilized to gather saponins from the specimen, and the overall saponin quantity was found by means of the vanillin-perchloric acid colorimetric method (Lu, 2022).

### 2.4 Data analysis

To obtain high-quality data, we utilized the DADA2, which includes steps such as primer trimming, quality filtering, denoising, merging, and chimera removal (Callahan et al., 2016). We employed the classify-sklearn algorithm in QIIME2 (version 2019.4)^1^ to annotate species for each amplicon sequence variants (ASV) feature sequence (Bokulich et al., 2018). Default parameters were used in QIIME2, and a pre-trained Naive Bayes classifier was utilized for the species annotation (DeSantis et al., 2006; Quast et al., 2012; Bolyen et al., 2019). To analyze the taxonomic composition and diversity of ZC larval gut microbiota, we used QIIME2 and executed the “qiime taxa barplot” command for taxonomic composition analysis. Using QIIME2 software and the ggplot2 package in R (version 4.2.1), we employed the “qiime diversity alpha-rarefaction” command with the following parameters: “--p-steps 10 --p-min-depth 10 --p-iterations 10.” This set the minimum rarefaction depth to 10, and the “--p-max-depth” parameter was set to 95% of the lowest sequencing depth among all samples. Subsequently, 10 evenly spaced depth values were selected between this depth and the minimum depth, with each depth value rarefied 10 times, and the selected alpha diversity indices were computed. The average score at the maximum rarefaction depth was chosen as the alpha diversity index. To visualize the alpha diversity differences between different sample groups, conduct a Kruskal-Wallis rank-sum test as a *post-hoc* test to confirm the significance of the differences, perform beta diversity analysis using the vegan package, and conduct PCA analysis using the ggplot2 package (Baselga and Orme, 2012; Lever et al., 2017; Villanueva and Chen, 2019). Functional prediction of ZC gut microbiota were predicted by PICRUSt2 (Phylogenetic investigation of communities by reconstruction of unobserved states) (Langille et al., 2013; Douglas et al., 2020) upon MetaCyc^2^ and KEGG^3^ databases.

To conduct network co-linearity analysis on gut microbiota, we used the interactive platform Gephi (v0.9.2), for network visualization (Jacomy et al., 2014). Nodes within the network reflect individual ASVs and edges represent pairwise correlations between nodes in the microbiota network. The computed features of the microbiota and fungal networks comprise positive co-occurrence and negative exclusion correlations, average path length, network diameter, average clustering coefficient, average connectivity, and modularity.

We executed a differential analysis using GraphPad Prism 9 to evaluate the disparities in the quantity of ZC on diverse varieties of pecan over five successive years, which yielded a violin plot (Mavrevski et al., 2018). To examine the PSMs content in various varieties of pecan on the OmicShare platform,^4^ we executed Kruskal-Wallis rank-sum tests and variance analysis. Alongside, we conducted Canonical Correspondence Analysis (CCA) on the ZC gut microbiota ASVs with weight adjustments ranging from 0.1 to 1. We also performed network weight analysis between ZC gut microbiota ASVs and PSMs of pecan. We performed a pathway analysis on ZC gut microbiota ASVs’ metabolic pathways and evaluated their correlation with the highly weighted ZC gut microbiota ASVs to determine the correlations between ZC gut microbiota ASVs on various pecan cultivars. We utilized the ImageGP platform^5^ to create a Manhattan plot.

## 3 Results

### 3.1 Differential analysis of ZC abundance on different varieties of pecan

**Figure 1:**
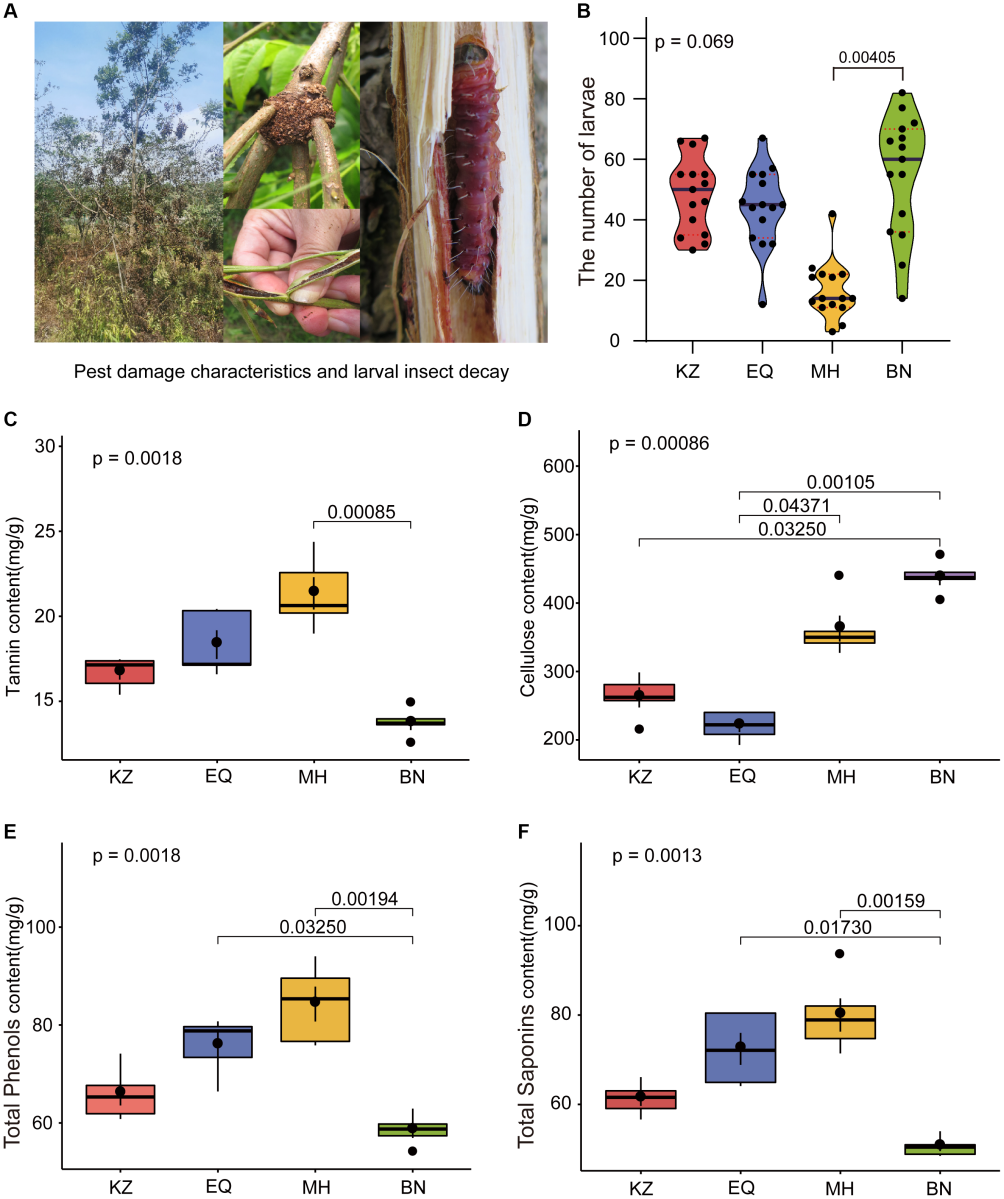
Statistical graphs of ZC larval damage rates and PSMs content among different pecan varieties. **(A)** The hotos depict the status of ZC larval damage. **(B)** The violin diagram shows the differential analysis of ZC survival on four varieties of pecan. The red dashed line represents the quartiles, the black line represents the median, and the black dots represent individual samples. **(C–F)** Differential analyses of tannin, cellulose, total phenols, and total saponin content in the branches of the four pecan varieties. The brackets and numerical values above the box plot indicate the difference in PSMs content between two pecan varieties.

Aimed to determine whether PSMs from four different varieties of pecan affect ZC population survival, the abundance of ZC living on these four varieties was statistically analyzed over 5 consecutive years (Figure 1B). It was observed that in the KZ variety, there were a total of 727 individuals over 5 years, there is an average of 48.467 ± 12.420 larvae per sampling point annually. In the EQ variety, there were 660 individuals, there is an average of 44.000 ± 13.196 larvae per sampling point annually. In the MH variety, there were 249 individuals, there is an average of 16.600 ± 9.387 larvae per sampling point annually. In the BN variety, there were 820 individuals, here is an average of 54.667 ± 20.013 larvae per sampling point annually. Notably, the number of ZC on the MH variety was lower than the other three varieties, and one-way ANOVA results indicated that MH was significantly lower than BN (*p* < 0.05).

### 3.2 Differential analysis of PSMs content in four pecan varieties

To further investigate a potential correlation between PSMs and ZC infestations, we analyzed the content of four insect-resistant PSMs in four different varieties of pecans (Figures 1C–F). Among the four varieties tested, MH had the highest tannin content (21.347 ± 2.217 mg/g), followed by EQ (18.329 ± 1.885 mg/g), KZ (16.683 ± 0.917 mg/g), and BN (13.688 ± 0.854 mg/g). Notably, BN had a significantly lower tannin content than MH (*p* < 0.05), and there were overall significant differences among the varieties (*p* < 0.001). The cellulose content varied significantly among the four varieties. BN had the highest content (436.732 ± 23.825 mg/g), followed by MH (362.653 ± 42.472 mg/g), KZ (262.139 ± 32.816 mg/g), and EQ (220.723 ± 20.714 mg/g), with EQ being significantly lower than MH and BN (*p* < 0.05), and KZ also significantly lower than BN (*p* < 0.05). Among the tested varieties, MH had the highest total phenol content (84.284 ± 7.095 mg/g), followed by EQ (75.798 ± 5.958 mg/g), KZ (65.057 ± 5.325 mg/g) and BN (58.475 ± 3.419 mg/g). BN had a significantly lower content in comparison to EQ and MH (*p* < 0.05). Additionally, significant differences among the varieties were observed overall (*p* < 0.05). Regarding total saponin content, the highest levels were found in MH (80.019 ± 8.303 mg/g), followed by EQ (72.432 ± 7.989 mg/g), KZ (61.288 ± 3.632 mg/g), and BN (50.547 ± 2.193 mg/g), with BN displaying significantly lower levels than EQ and MH (*p* < 0.05), and statistically significant differences among the varieties overall (*p* < 0.05). It was determined that, excluding cellulose, MH exhibited lower levels than BN in other PSMs, while the remaining varieties displayed higher levels than other varieties.

### 3.3 Differential analysis of ZC gut microbiota composition

**Figure 2:**
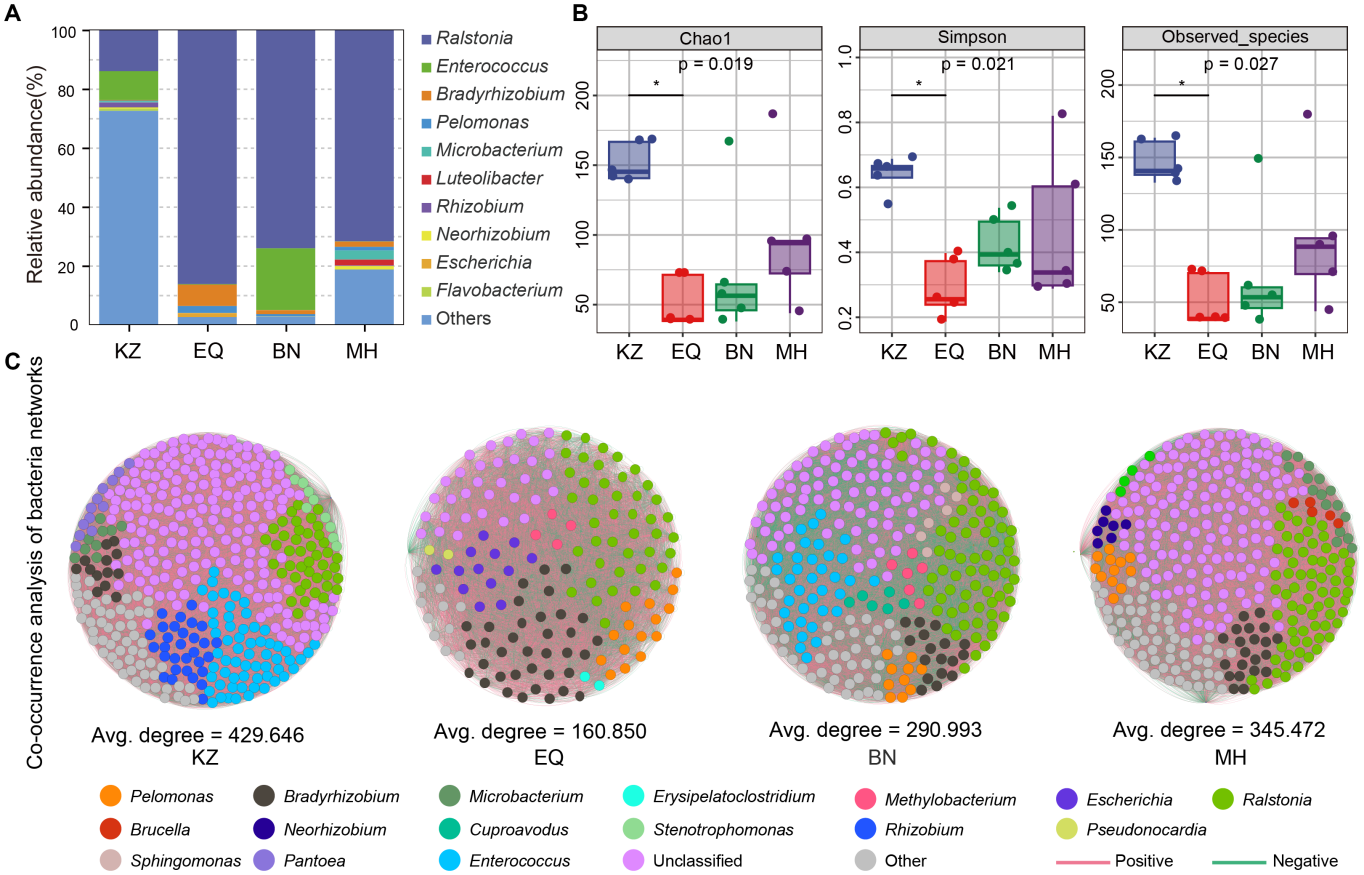
Intestinal microbiota species composition, diversity, and network co-linearity analysis. **(A)** The proportional histogram presents the analysis of the top 10 abundant genera-level species compositions in the ZC gut microbiota. **(B)** The box diagrams display the alpha diversity analysis of ZC gut microbiota, including Chao1, Simpson, and Observed_species analyses. The top and bottom lines of the box represent the upper and lower quartiles. The line inside the box represents the median. The upper and lower edges of the box represent the maximum and minimum values. Points outside the upper and lower edges of the box represent outliers. The numbers under the diversity index labels indicate the *p*-values from the Kruskal-Wallis test. When conducting pairwise comparisons, ^⁎^ marks are used as default significance markers. **(C)** Co-linearity analysis of gut microbiota networks in different pecan varieties for ZC larvae. Each point represents an ASV, with the point’s size indicating its proportion among all ASVs. Red lines represent positive correlations, while green lines indicate negative correlations.

After quality control of the sequencing raw data, ZC larval gut samples from the KZ, EQ, BN, and MH groups yielded a total of 126,169, 103,487, 123,267, and 115,856 effective microbiota sequences, respectively. Dilution curve analysis (Supplementary Figure S1) showed that as the sequencing depth increased, the number of ASVs in ZC gut microbiota tended to stabilize, indicating that the sequencing depth of the samples met the requirements for diversity analysis (Supplementary Figure S1). Analysis of the gut microbiota community composition of ZC larvae feeding on four different pecan varieties (Figure 2A) revealed the following proportions of genera that make up more than 1% of the composition: In the KZ variety: *Ralstonia* (14.02%), *Enterococcus* (9.83%), and *Rhizobium* (1.64%). In the EQ variety: *Ralstonia* (86.28%), *Bradyrhizobium* (7.28%), *Pelomonas* (2.37%), and *Escherichia* (1.35%). In the BN variety: *Ralstonia* (74.15%), *Enterococcus* (20.94%), and *Bradyrhizobium* (1.38%). In the MH variety: *Ralstonia* (71.79%), *Microbacterium* (3.11%), *Luteolibacter* (1.94%), *Bradyrhizobium* (1.82%), *Neorhizobium* (1.20%), and *Pelomonas* (1.20%).

The alpha diversity analysis of ZC gut microbiota is mainly based on Chao1, Simpson, and Observed_species indices (Figure 2B) (Chao, 1984). The Chao1 index, which measures species richness, indicates that the gut microbiota richness of the KZ variety was significantly higher than that of other varieties, with a statistically significant difference (*p* < 0.05) (Figure 2B). The Simpson index demonstrated that the KZ variety housed the highest gut microbiota diversity and evenness, significantly surpassing other varieties (*p* < 0.05) (Figure 2B) (Simpson, 1949). The Observed_species richness index quantified the number of ASVs detected in the sample, revealing that the KZ variety had significantly higher gut microbiota ASV richness than other host plants (*p* < 0.05) (Figure 2B).

To investigate the interactions among different microbiota, a co-occurrence network analysis was performed on the ZC larval gut microbiota (Figure 2C; Table 1). The network diagrams illustrate the correlations between the ZC microbiota in each variety (Figure 2C), with KZ, EQ, BN, and MH including 429, 160, 290, and 345 nodes, respectively. The number of positive correlations in the KZ, EQ, BN, and MH groups were 34,399, 5,110, 17,589, and 22,637, respectively. The data revealed 57,805, 7,764, 24,605, and 37,025 negative correlations among microbiota communities. A community co-occurrence positive/negative link ratio less than 1 for all strains demonstrates a preponderance of negative correlations (Table 1), inferring antagonistic relations among microbiota communities.

**Table 1:** Characteristics of the co-linearity network of ZC larval gut microbiota.

ID	Node	Positive edge	Negative edge	Average weighted degree	Modularity	Average clustering coefficient	Average path distance
KZ	429	34,388	57,805	14.019	8.967	0.999	1.001
EQ	160	5,110	7,764	18.975	2.648	0.997	1.001
BN	290	17,589	24,605	110.458	0.433	0.999	1.000
MH	345	22,637	37,025	87.149	0.818	0.998	1.002

**Figure 3:**
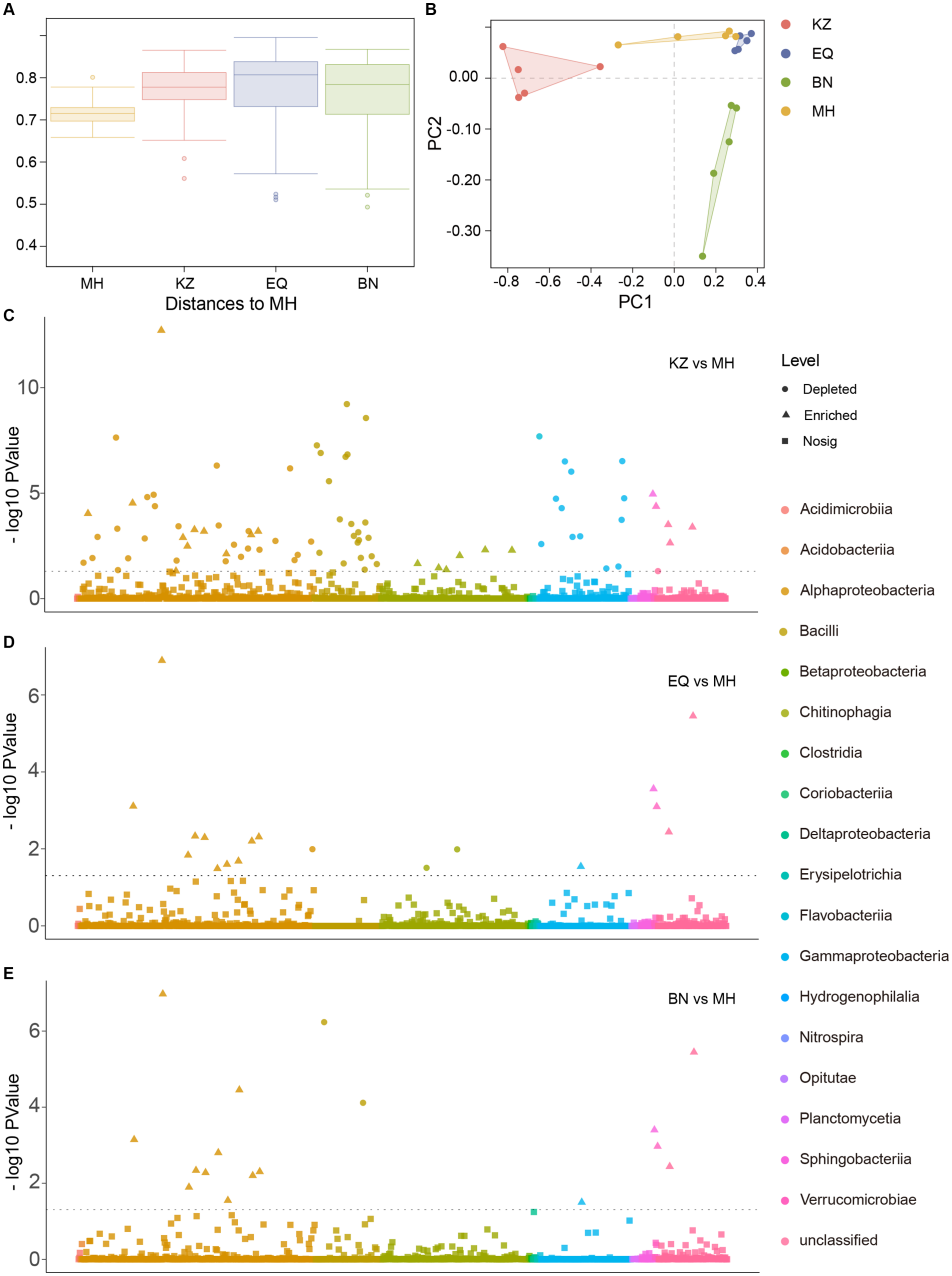
Inter-group ASV differential analysis. **(A)** Beta diversity analysis of ZC larval gut microbiota. In the box plot, the top and bottom lines of the box represent the upper and lower quartiles. The line inside the box represents the median. The upper and lower edges of the box represent the maximum and minimum values. Points outside the upper and lower edges of the box represent outliers. **(B)** The Principal Component Analysis (PCA) of midgut microbiota concerning ZC. **(C–E)** is a Manhattan plot illustrating the differential analysis of ASVs in the gut microbiota of ZC larvae between the KZ, EQ, and BN varieties compared to the MH variety. The horizontal line represents the *p*-value threshold line, typically indicating the significance level threshold for better understanding the significance level of each data point. In the “Level” category, circular symbols represent “depleted,” triangular symbols indicate “significantly enriched,” and square symbols represent “no significant change.”

Beta diversity analysis was performed on the gut microbiota of ZC from four pecan varieties (Figure 3A; Supplementary Table S1). Results of the permdisp test showed a significant difference (*p* < 0.05) between the pecans of the MH variety and the KZ variety. On the other hand, the differences between the EQ and MH varieties and between the BN and MH varieties were not significant, with *p*-values of 0.621 and 0.634, respectively. The evidence suggests that the gut microbiota of ZC on BN, EQ, and MH varieties exhibit greater similarity. Furthermore, Principal Component Analysis (PCA) showed that the gut microbiota community structures of ZC larvae on KZ, BN, EQ, and MH varieties of pecan are distinct (Figure 3B). The gut microbiota community structures of ZC on EQ and MH cultivars cluster together and are distinct from those on KZ and BN cultivars, suggesting a similarity between the microbiota community structures of EQ and MH cultivars and dissimilarities from the other two cultivars.

To determine the distinct dissimilarities among the ASVs of ZC larval gut microbiota on the KZ, BN, and EQ varieties of pecans in comparison to MH, a correlation analysis was conducted (Figures 3C–E). The examination exposed significant associations between the ZC gut microbiota ASVs on the three pecan varieties and the ASVs on the MH (Supplementary Tables S2–S4). There were 83 ASVs that differed significantly between KZ and MH groups, 18 ASVs that differed significantly between EQ and MH, and 17 ASVs that differed significantly between BN and MH. These findings indicate that the microbiota ASV structures in EQ, BN, and MH varieties are similar, with fewer differences observed when compared to KZ and MH varieties.

### 3.4 Differential analysis of PSMs content and ASVs of ZC gut microbiota

**Figure 4:**
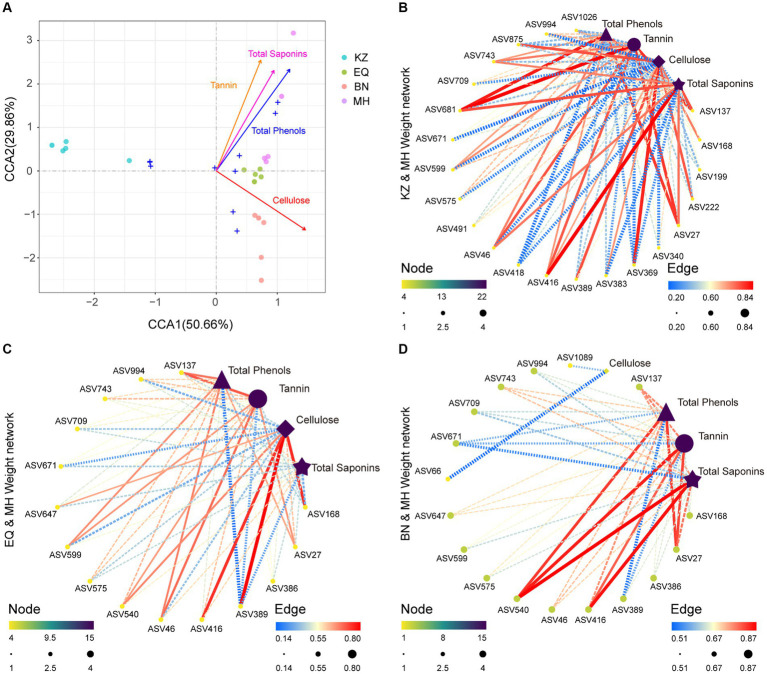
Correlation analysis between microbiota and environmental factors. **(A)** CCA plot illustrating the relationship between all ZC gut microbiota ASVs and PSMs. Different colored circles represent the varying PSM content in samples from different pecan varieties. **(B–D)** Correlation network plots between ZC gut microbiota ASVs that significantly differ in the KZ, EQ, and BN pecan varieties compared to the MH variety and their corresponding PSMs. The size and color of the points, transitioning from yellow to green to purple, signify the significance of each data point. The thickness and color of the lines, spanning from blue to yellow to red, represent the magnitude of the correlation.

To determine whether the PSMs of pecans are key factors influencing the structure of ZC gut microbiota, a correlation analysis was conducted (Figure 4A). The analysis revealed positive correlations between tannin, total saponins, total phenols, and gut microbiota, and a negative correlation with cellulose (Figures 4B–D). Based on the results of differential analysis of gut microbiota ASVs between the MH variety and the other three varieties, a correlation analysis was performed between the significantly different ASVs in each group and the PSMs of pecan in each group. This analysis determined the strength of the correlation between ZC gut microbiota ASVs with significant differences and the PSMs of pecan (tannin, total phenols, cellulose, and total saponins) between the two samples (Figures 4B–D).

The analysis indicates that in KZ, BN, EQ, and MH groups, specific enriched ASVs, including ASV46 (*Pararhizobium* sp.), ASV994 (*Olivibacter* sp.), ASV743 (*Rhizobium* sp.), ASV709 (*Rhizobium* sp.), ASV671 (*Luteolibacter* sp.), ASV599 (*Agrobacterium* sp.), ASV575 (*Microbacterium* sp.), and ASV27 (*Rhizobium* sp.), are positively correlated with pecan PSMs tannin, total saponins, and total phenols, while being significantly inhibited by pecan PSMs cellulose. ASV389 (*Brevundimonas* sp.) and ASV168 (*Schumannella* sp.) are positively correlated with pecan PSMs cellulose and are significantly inhibited by pecan PSMs tannin, total saponins, and total phenols. ASV416 (*Falsirhodobacter* sp.) is positively correlated with pecan PSMs cellulose, tannin, total saponins, and total phenols (Figures 4B–D).

### 3.5 Functional prediction of ZC gut microbiota

**Figure 5:**
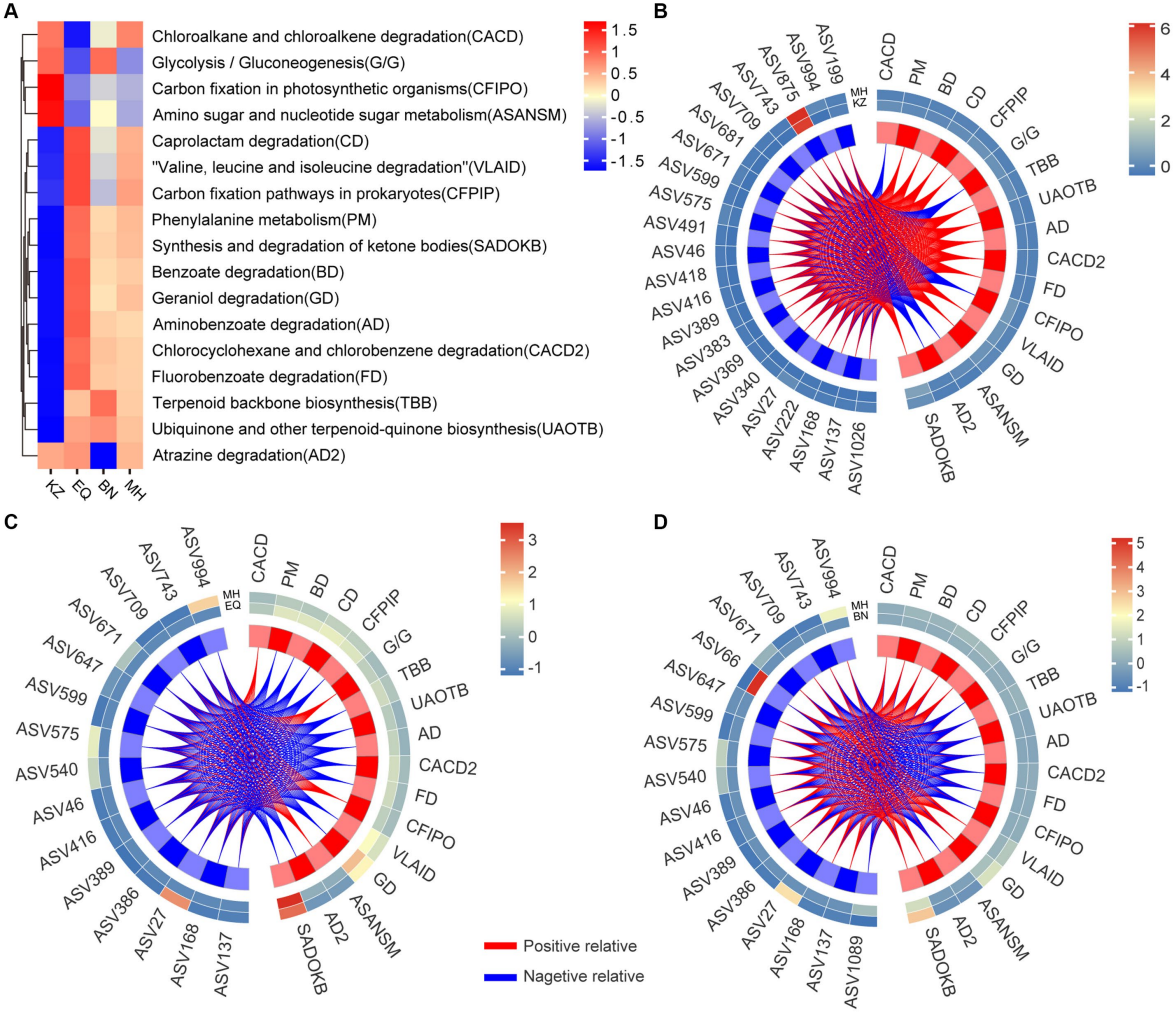
Analysis of the correlation between specificity of gut strains and gut microbiota functionality. **(A)** The heat map of metabolic pathways for ZC gut microbiota across four pecan varieties is displayed, showing the differences in pathway activities. The color gradient from blue to yellow to red illustrates the transition of metabolic functions within ZC larval gut ASVs from a negative to a positive trend across distinct pecan varieties. **(B–D)** Correlation heat maps are visual representations depicting the networks between ZC gut microbiota ASVs that significantly differ in the KZ, EQ, and BN pecan varieties compared to the MH variety and their association with metabolic pathways. The colors on the heat map represent the magnitude of correlation between the two varieties, transitioning from blue to red. The red lines represent positive correlations, while the blue lines represent negative correlations.

Functional prediction of metabolic pathways between different groups (Figure 5A) may reveal distinct differences in metabolic pathways between the KZ variety and EQ, BN, and MH varieties. Additionally, EQ, BN, and MH varieties showed similar metabolic pathways, indicating that the gut microbiota community structure in these three varieties might possess similar decomposition abilities, while the KZ variety differs in metabolic capabilities from the other three varieties. Through the correlation analysis of metabolic pathways and microbiota ASVs (Figures 5B–D; Supplementary Tables S5–S7), it was observed that the KZ variety exhibited 264 positive correlations and 110 negative correlations with metabolic pathways compared to the MH variety. The EQ variety showed 60 positive correlations and 195 negative correlations with metabolic pathways compared to the MH variety. The BN variety displayed 151 positive correlations and 138 negative correlations with metabolic pathways compared to the MH variety. These results suggest that the differences in metabolic pathways between the KZ and MH varieties are positively correlated with the gut microbiota, while the differences in metabolic pathways between the EQ and MH varieties are negatively correlated, and the differences in metabolic pathways between the BN and MH varieties are positively correlated.

Furthermore, an analysis of the specific enriched ASVs, including ASV46, ASV994, ASV743, ASV709, ASV671, ASV599, ASV575, and ASV27, revealed the following patterns: In the KZ and MH varieties, these ASVs were associated with 17 metabolic pathways. Out of these, 12 pathways exhibited positive correlations, including Phenylalanine metabolism (PM), Benzoate degradation (BD), Caprolactam degradation (CD), Carbon fixation pathways in prokaryotes (CFPIP), Terpenoid backbone biosynthesis (TBB), Ubiquinone and other terpenoid-quinone biosynthesis (UAOTB), Aminobenzoate degradation (AD), Chlorocyclohexane and chlorobenzene degradation (CACD2), Fluorobenzoate degradation (FD), Valine, leucine and isoleucine degradation (VLAID), Geraniol degradation (GD), and Synthesis and degradation of ketone bodies (SADOKB). Additionally, 5 pathways exhibited negative correlations, including Chloroalkane and chloroalkene degradation (CACD), Glycolysis / Gluconeogenesis (G/G), Carbon fixation in photosynthetic organisms (CFIPO), Amino sugar and nucleotide sugar metabolism (ASANSM), and Atrazine degradation (AD2). In the BN and MH varieties, these ASVs were associated with 17 metabolic pathways as well. Among them, 9 pathways showed positive correlations, including CACD, PM, BD, CD, CFPIP, VLAID, GD, AD2, and SADOKB, while 8 pathways showed negative correlations, including G/G, TBB, UAOTB, AD, CACD2, FD, CFIPO, and ASANSM. In the EQ and MH varieties, these ASVs were linked to 17 metabolic pathways. Four pathways had positive correlations, namely CACD, G/G, CFIPO, and ASANSM, while 13 pathways exhibited negative correlations, including PM, BD, CD, CFPIP, TBB, UAOTB, AD, CACD2, FD, VLAID, GD, AD2, and SADOKB.

## 4 Discussion

Plant secondary metabolites (PSMs) resulting from plant and insect co-evolution are often toxic to phytophagous insects feeding on different host plants, impacting their growth and development (Janson et al., 2008; Mason et al., 2018, 2019; Mason, 2020). The completion of the phytophagous insects’ life cycle is heavily dependent on a favorable host environment (Kolodny et al., 2020). The PSMs also function as pest-resistance agents (Zhang et al., 2023). Insects face strong selection pressures from defensive chemicals when they feed on unsuitable hosts or change host plants (Shikano, 2017). *Papilio polytes* exhibit different feeding, growth, and reproduction habits depending on their host plants (Shobana et al., 2010). *Epilachna dodecastigma* larvae have different life cycles that are influenced by nutrient levels and PSMs and are sustained by young, mature, and aged *Momordica charantia* leaves (Sarkar et al., 2016). Insects can survive in nature with some degree of success if they consume plant matter that is free of poison. This study found variations in the damage caused by ZC larvae among different pecan varieties. The MH variety had the lowest number of ZC larvae, while the BN variety had the highest. This difference may be related to the varying levels of PSMs in each variety, but it is unclear which specific PSM is responsible for the anti-insect effect (Figure 1).

Previous research has shown that *Spilosoma obliqua* survival rates were lower on varieties of *Vigna radiata* with higher PSM content, while higher on varieties with lower PSM content. This finding is consistent with that of Mobarak et al. (2020). Changes in the gut microbiota structure can enhance insects’ ability to feed on and digest plant hosts (Feldhaar, 2011; Engel and Moran, 2013; Chen et al., 2016). They can also help hosts adapt and defend against metabolic chemicals (Feldhaar, 2011; Engel and Moran, 2013; Chen et al., 2016). However, sudden increases in PSM concentration can continuously impact the microbiotas involved in the intestinal nutrient metabolism of herbivorous insects during the digestion of plant material. An increase in plant secondary metabolites (PSMs) can cause insects to become malnourished or even suffer from toxicity if they cannot properly digest nutrients (Mason et al., 2018, 2019; Mason, 2020). It is important to note that this effect can occur due to external factors such as the use of pesticides. Our study suggests that the low number of MH clones may be caused by the high content of PSMs. The larvae had a low diversity of gut microbiome due to the high content of PSMs (Figures 2A,B). At the meantime, the network diagram analysis revealed a higher negative correlation between the number of ASVs in intestinal samples from MH and KZ (Figure 2C; Table 1). This also suggests that eating plants with high PSMs content may negatively regulate the intraspecific interrelationships of gut microbiota (Zhang et al., 2022 2023). The CCA analysis also showed that the three resistant PSMs had similar effects on the gut microbiota structure (Figure 4A). Thus, we believed that tannin, total phenols, and total saponins were the key secondary metabolites related to insect resistance in pecans. Additionally, cellulose may provide nutrients for ZC larvae.

Like certain compounds that affect the nervous or endocrine systems, plant PSMs target microbiota upon entering the gut, subsequently affecting insect growth and development (Ceja-Navarro et al., 2015). The microbiota functions as an essential virtual digestive system for herbivorous insects (Hammer and Bowers, 2015; Zhang et al., 2018). The microbiota in the insect midgut is crucial for the effects of (3E)-4,8-dimethyl-1,3,7-nonatriene on the structure of the peritrophic matrix and the elimination of insect pests (Chen et al., 2021). Additionally, numerous studies have confirmed the bactericidal properties of these metabolites. For instance, the quantity of tannins present in the host plant *Triadica sebifera* has a significant inverse correlation with the survival of a specific herbivorous insect known as *Bikasha collaris* (Huang et al., 2014). Similarly, tea saponins impede the gut microbiota responsible for nutrient digestion in *Curculio chinensis*, resulting in a decrease in the survival rate (Zhang H. et al., 2020; Zhang S. et al., 2020). Rice with increased levels of flavonoids and total phenols can hinder the reproductive ability of *Sogatella furcifera*, according to a study by Hu et al. (2023). This study also found significant variations in the ZC gut microbiota structure among different pecan varieties. The intestinal tracts of PSMs contained high concentrations of *Pararhizobium* sp. (ASV46), *Olivibacter* sp. (ASV994), *Rhizobium* sp. (ASV743 and ASV709), *Luteolibacter* sp. (ASV671), *Agrobacterium* sp. (ASV599), *Microbacterium* sp. (ASV575), and *Rhizobium* sp. (ASV27). These microbiotas were found to be associated with the metabolism of toxic substances (Figure 5). Previous studies have demonstrated that *Olivibacter* bacteria can break down phenolic substances, while Rhizobium bacteria are capable of degrading flavonoids (Ntougias et al., 2014; Cotton et al., 2019). The abundance of *Brevundimonas* sp. (ASV389) and *Schumannella* sp. (ASV168), which are associated with nutritional digestion, is relatively low (Figure 5). The findings and illustrations support the idea that the presence of PSMs affects the gut microbiota related to nutrient digestion, which in turn hinders nutrient availability for plant-feeding insects (Yonekura-Sakakibara et al., 2019; Li et al., 2022; Phukan et al., 2023). The dysbiosis of the gut microbiota negatively impacts the survival of ZC larvae. When the level of PSM concentration exceeds a certain threshold, it can lead to the loss of functional bacteria in the intestinal tract of ZC larvae. These bacteria assist in nutrition metabolism, and their loss can cause malnutrition and prevent the larvae from completing their life cycle. The damage rates of ZC larvae vary across different host plant genera due to differences in PSM content within various pecan species, as evidenced by variations in population and gut microbiota.

## 5 Conclusion

Our study examined the effects of differing secondary metabolite levels in various pecan varieties on the gut microbiota structure of ZC larvae, with specific attention to tannins, cellulose, total phenols, and total saponins. The results showed that the diversity of ZC gut microbiota was significantly affected by the levels of PSMs in pecan varieties, resulting in variations in gut microbiota community structures. Furthermore, the study revealed connections between crucial metabolic pathways and critical ASVs in the ZC gut. This research clarifies the significance of PSMs, like tannins, total phenols, and total saponins, in resisting insects by providing theoretical and technical support for breeding insect-resistant pecan varieties and developing new environmentally friendly methods to manage ZC.

## Data availability statement

The datasets presented in this study can be found in online repositories. The names of the repository/repositories and accession number(s) can be found in the article/Supplementary material.

## Author contributions

JW: Writing – original draft. SZ: Writing – review & editing. JK: Writing – original draft, Software, Formal analysis, Data curation. JC: Writing – original draft, Funding acquisition.

## Footnotes

^1^ https://github.com/QIIME2/q2-feature-classifier

^2^ https://metacyc.org/

^3^ https://www.kegg.jp/

^4^ https://www.genedenovo.com/

^5^ https://www.bic.ac.cn/ImageGP
