# Supplementary material for: Pecan secondary metabolites influenced the population of Zeuzera coffeae by affecting the structure and function of the larval gut microbiota
Source: Front Microbiol. 2024 Apr 12;15:1379488. doi: 10.3389/fmicb.2024.1379488 (PMC11045946; doi:10.3389/fmicb.2024.1379488)
Supplement: Supplementary file 1 [file Data_Sheet_1.PDF]

Pecan secondary metabolites influenced the population of *Zeuzera coffeae* by affecting the structure and function of the larval gut microbiota

Jie Wang[1], Shouke Zhang[1,*], Junqia Kong[2,*], Jun Chang[3,*]

[1]State Key Laboratory of Subtropical Silviculture, Zhejiang A&F University, Hangzhou Zhejiang 311300, People's Republic of China

[2]College of Landscape Architecture, Zhejiang A&F University, Hangzhou Zhejiang 311300, People's Republic of China

[3]Research Institute of Subtropical Forestry, Chinese Academy of Forestry, Hangzhou Zhejiang 311400, People's Republic of China

*Correspondence to: Shouke Zhang, Email: socrazhang@zafu.edu.cn;
Junqia Kong, Email: kongjunqia@lzb.ac.cn;
Jun Chang, Email: ylschj@caf.ac.cn.

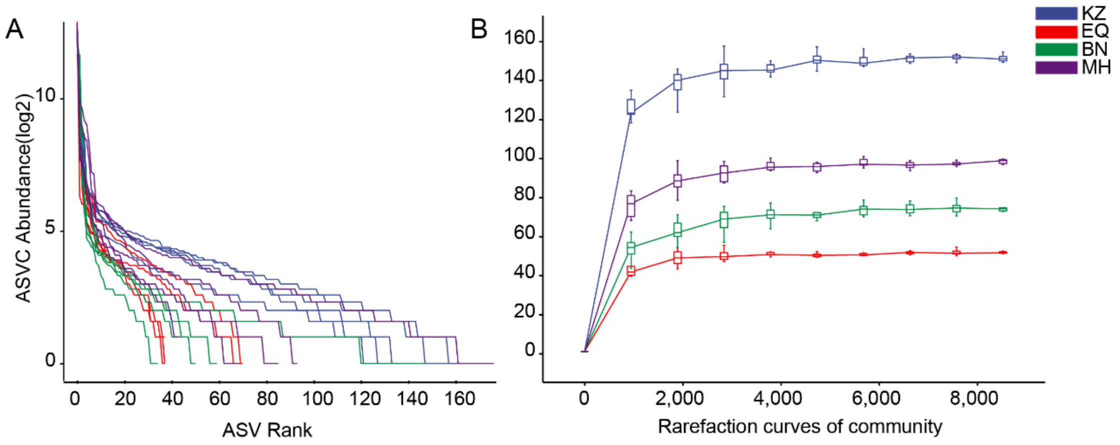

Fig. S1. Bacterial rarefaction curves are used to assess the depth of coverage for multi samples. (A)Rank aboundance. (B) Rarefaction curves of community
(Multi samples were distinguished by different colors of lines)

Table S1. Analysis of beta diversity index between groups

Group1	Group2	Sample#size	Permutations	F#value	p#value
KZ	MH	10	999	2.269142	0.042
EQ	MH	10	999	0.147446	0.621
BN	MH	10	999	0.141095	0.634

Table S2 The differences of strains between different samples were compared（KZ VS MH）

KZ VS MH					
ID	log2FC	PValue	FDR	level	Phylum
ASV1005	-20.24518593	0.002583011	0.033871555	Depleted	Proteobacteria
ASV101	-19.97669557	0.019854132	0.191647526	Depleted	Proteobacteria
ASV1015	-18.68719112	0.04979403	0.416950013	Depleted	Actinobacteria
ASV1026	5.456908659	9.27E-05	0.002575881	Enriched	Proteobacteria
ASV1035	-22.30516667	5.44E-08	6.30E-06	Depleted	Firmicutes
ASV1058	-20.58335276	0.011915304	0.123599051	Depleted	Proteobacteria
ASV106	-19.64654816	0.006702346	0.077635503	Depleted	Firmicutes
ASV1067	-22.15388844	1.25E-07	1.24E-05	Depleted	Firmicutes
ASV1083	-19.5172657	0.001187242	0.018753033	Depleted	Proteobacteria
ASV1147	-22.38145001	2.32E-08	3.23E-06	Depleted	Proteobacteria
ASV1150	-21.18968945	0.000484913	0.009912191	Depleted	Proteobacteria
ASV1151	-19.32462007	0.044446383	0.381360936	Depleted	Proteobacteria
ASV1166	-20.99299779	1.84E-05	0.000639496	Depleted	Proteobacteria
ASV121	-20.49454507	0.012257854	0.125282483	Depleted	Proteobacteria
ASV137	21.7651922	2.96E-05	0.000980388	Enriched	Proteobacteria
ASV148	-21.37524523	2.72E-06	0.000126232	Depleted	Firmicutes
ASV156	-20.92665505	5.15E-05	0.001491204	Depleted	Proteobacteria
ASV168	21.50594817	0.000311962	0.007227124	Enriched	Actinobacteria
ASV190	-19.5172657	0.001406882	0.020803893	Depleted	Proteobacteria
ASV199	3.337871682	0.002304374	0.030798847	Enriched	0
ASV2	-20.85711385	1.54E-05	0.000594902	Depleted	Proteobacteria
ASV204	-21.75566919	3.15E-07	1.99E-05	Depleted	Proteobacteria
ASV222	2.16766563	0.022032224	0.20692427	Enriched	Proteobacteria
ASV233	-20.87481779	1.19E-05	0.000487339	Depleted	Proteobacteria
ASV235	-20.89230267	4.15E-05	0.001284298	Depleted	Proteobacteria
ASV257	-21.83919225	9.59E-07	4.76E-05	Depleted	Proteobacteria
ASV26	-20.78405009	0.001185508	0.018753033	Depleted	Proteobacteria
ASV27	25.59140535	1.92E-13	1.34E-10	Enriched	Proteobacteria
ASV340	19.32462007	0.049200731	0.416950013	Enriched	Proteobacteria
ASV344	-20.24518361	0.015755069	0.156425324	Depleted	Proteobacteria
ASV35	-21.72671709	0.000174996	0.004677767	Depleted	Firmicutes
ASV352	-21.2986231	0.000372198	0.008083677	Depleted	Proteobacteria
ASV369	5.380827362	0.001272798	0.019657657	Enriched	Proteobacteria
ASV37	-19.94353072	0.021495799	0.204651786	Depleted	Firmicutes
ASV382	-21.77465196	1.90E-07	1.46E-05	Depleted	Firmicutes
ASV383	19.47146089	0.034814987	0.314239166	Enriched	Proteobacteria
ASV386	-4.568939586	0.001111813	0.018397855	Depleted	Proteobacteria
ASV389	20.24518361	0.003265641	0.041265827	Enriched	Proteobacteria
ASV416	21.25873049	0.000538068	0.010684501	Enriched	Proteobacteria
ASV418	19.47145692	0.042703784	0.370989126	Enriched	Proteobacteria
ASV419	-23.16831557	6.02E-10	2.09E-07	Depleted	Firmicutes

ASV434	-21.80266624	1.47E-07	1.27E-05	Depleted	Firmicutes
ASV46	21.43612591	0.000641152	0.011803463	Enriched	Proteobacteria
ASV491	4.149741487	0.009189679	0.101378209	Enriched	Proteobacteria
ASV492	-20.00911785	0.000289435	0.006936456	Depleted	Firmicutes
ASV540	-4.598385909	4.95E-07	2.87E-05	Depleted	Proteobacteria
ASV558	-20.83919302	0.00106411	0.018037962	Depleted	Firmicutes
ASV56	-5.297685067	0.000340983	0.007644619	Depleted	Proteobacteria
ASV575	3.56358972	0.000407497	0.008582136	Enriched	Actinobacteria
ASV596	-19.21770216	0.016955227	0.165970181	Depleted	Proteobacteria
ASV599	21.24518477	0.007525644	0.085742993	Enriched	Proteobacteria
ASV604	-20.42415398	0.002207114	0.030077342	Depleted	Firmicutes
ASV608	-6.34089709	0.000715044	0.012742459	Depleted	Firmicutes
ASV610	-20.51726377	0.001695869	0.024554776	Depleted	Firmicutes
ASV635	-20.24518593	0.002776909	0.03573985	Depleted	Proteobacteria
ASV646	-20.58335276	0.011706321	0.123271103	Depleted	Firmicutes
ASV662	-20.68718942	0.010447817	0.111711275	Depleted	Proteobacteria
ASV671	24.14844089	4.25E-05	0.001284298	Enriched	Verrucomicrobia
ASV68	-22.61529601	2.04E-08	3.23E-06	Depleted	Bacteroidetes
ASV681	19.80266466	0.004905274	0.058778715	Enriched	Proteobacteria
ASV698	-19.87481629	0.004242691	0.052654831	Depleted	Proteobacteria
ASV7	-19.80266466	0.000631669	0.011803463	Depleted	Proteobacteria
ASV709	21.21770334	0.000940553	0.016342102	Enriched	Proteobacteria
ASV730	-20.72671709	0.042278309	0.370989126	Depleted	Firmicutes
ASV736	-20.24518593	0.000246144	0.00610964	Depleted	Firmicutes
ASV740	-22.7267175	2.75E-09	6.37E-07	Depleted	Firmicutes
ASV743	21.28544737	0.000645369	0.011803463	Enriched	Proteobacteria
ASV752	-21.37524523	0.004799085	0.058515157	Depleted	Proteobacteria
ASV770	-19.04083016	0.037250837	0.331914513	Depleted	Proteobacteria
ASV772	-20.70708778	0.001314736	0.019863944	Depleted	Firmicutes
ASV814	-19.64654816	0.00184254	0.026133985	Depleted	Proteobacteria
ASV820	-20.78404849	0.009839682	0.106852798	Depleted	Firmicutes
ASV875	2.370610029	0.005106029	0.060147293	Enriched	Proteobacteria
ASV884	-21.70708778	6.72E-07	3.59E-05	Depleted	Proteobacteria
ASV893	-19.04082481	0.030099437	0.275251429	Depleted	Proteobacteria
ASV901	-20.27215135	0.014975632	0.150841506	Depleted	Proteobacteria
ASV904	-19.83919456	0.023014229	0.213265192	Depleted	Firmicutes
ASV917	-3.932883107	0.008417095	0.094352922	Depleted	Proteobacteria
ASV918	-20.94352929	0.000184324	0.004744649	Depleted	Proteobacteria
ASV924	-21.69717375	3.04E-07	1.99E-05	Depleted	Proteobacteria
ASV942	-20.9095811	1.75E-05	0.000639496	Depleted	Proteobacteria
ASV981	-19.64654816	0.001982103	0.027551226	Depleted	Proteobacteria
ASV994	25.20638834	1.11E-05	0.000483993	Enriched	Bacteroidetes

Table S3 The differences of strains between different samples were compared（EQ VS MH）

EQ VS MH					
ID	log2FC	PValue	FDR	level	Phylum
ASV137	21.7651922	0.000777778	0.070116301	Enriched	Proteobacteria
ASV168	21.50594817	0.003635195	0.246575161	Enriched	Actinobacteria
ASV265	-20.72671875	0.031059007	0.793328031	Depleted	Proteobacteria
ASV27	25.59140535	1.28E-07	5.64E-05	Enriched	Proteobacteria
ASV386	20.89230415	0.028652837	0.786162205	Enriched	Proteobacteria
ASV389	20.24518361	0.014563396	0.49179468	Enriched	Proteobacteria
ASV416	21.25873049	0.004638521	0.246575161	Enriched	Proteobacteria
ASV46	21.43612591	0.005055072	0.246575161	Enriched	Proteobacteria
ASV474	-20.60472876	0.010298628	0.376758128	Depleted	Proteobacteria
ASV540	3.891013064	0.032528256	0.793328031	Enriched	Proteobacteria
ASV575	24.75327797	3.53E-06	0.000774241	Enriched	Actinobacteria
ASV599	21.24518477	0.025162724	0.736429068	Enriched	Proteobacteria
ASV647	5.197708862	0.020731925	0.65009392	Enriched	Proteobacteria
ASV671	24.14844089	0.000798591	0.070116301	Enriched	Verrucomicrobia
ASV709	21.21770334	0.006298626	0.276509674	Enriched	Proteobacteria
ASV743	21.28544737	0.004895039	0.246575161	Enriched	Proteobacteria
ASV99	-4.735322678	0.010191435	0.376758128	Depleted	Proteobacteria
ASV994	25.20638834	0.000274981	0.040238873	Enriched	Bacteroidetes

Table S4 The differences of strains between different samples were compared（BNVS MH）

BN VS MH					
ID	log2FC	PValue	FDR	level	Phylum
ASV1089	-24.6375017	0.000000583	0.00016128	Depleted	Firmicutes
ASV137	21.7651922	0.000713229	0.056345066	Enriched	Proteobacteria
ASV168	21.50594817	0.003639253	0.201250694	Enriched	Actinobacteria
ASV27	25.59140535	0.000000106	0.0000584	Enriched	Proteobacteria
ASV386	20.89230415	0.031716807	1	Enriched	Proteobacteria
ASV389	20.24518361	0.012722837	0.469048591	Enriched	Proteobacteria
ASV416	21.25873049	0.004600709	0.224232246	Enriched	Proteobacteria
ASV46	21.43612591	0.005271282	0.224232246	Enriched	Proteobacteria
ASV540	7.372144039	0.001575953	0.09683355	Enriched	Proteobacteria
ASV575	24.75327797	0.00000359	0.000662185	Enriched	Actinobacteria
ASV599	21.24518477	0.028284596	0.97758636	Enriched	Proteobacteria
ASV647	23.03690035	0.0000353	0.004882495	Enriched	Proteobacteria
ASV66	-27.1169443	0.0000769	0.008505087	Depleted	Firmicutes
ASV671	24.14844089	0.001075082	0.074315023	Enriched	Verrucomicrobia
ASV709	21.21770334	0.006306506	0.249106971	Enriched	Proteobacteria
ASV743	21.28544737	0.004966129	0.224232246	Enriched	Proteobacteria
ASV994	25.20638834	0.000397999	0.036682275	Enriched	Bacteroidetes

Table S5 Correlation analysis between different strains and metabolic function（KZ&MH&Pathway）

KZ&MH&Pathway		
Var1	Var2	cor
ASV1026	Chloroalkane and chloroalkene degradation	-1
ASV137	Chloroalkane and chloroalkene degradation	-1
ASV168	Chloroalkane and chloroalkene degradation	-1
ASV222	Chloroalkane and chloroalkene degradation	-1
ASV27	Chloroalkane and chloroalkene degradation	-1
ASV340	Chloroalkane and chloroalkene degradation	-1
ASV369	Chloroalkane and chloroalkene degradation	-1
ASV383	Chloroalkane and chloroalkene degradation	-1
ASV389	Chloroalkane and chloroalkene degradation	-1
ASV416	Chloroalkane and chloroalkene degradation	-1
ASV418	Chloroalkane and chloroalkene degradation	-1
ASV46	Chloroalkane and chloroalkene degradation	-1
ASV491	Chloroalkane and chloroalkene degradation	-1
ASV575	Chloroalkane and chloroalkene degradation	-1
ASV599	Chloroalkane and chloroalkene degradation	-1
ASV671	Chloroalkane and chloroalkene degradation	-1
ASV681	Chloroalkane and chloroalkene degradation	-1
ASV709	Chloroalkane and chloroalkene degradation	-1
ASV743	Chloroalkane and chloroalkene degradation	-1
ASV875	Chloroalkane and chloroalkene degradation	-1
ASV994	Chloroalkane and chloroalkene degradation	-1
ASV199	Chloroalkane and chloroalkene degradation	-1
ASV1026	Phenylalanine metabolism	1
ASV137	Phenylalanine metabolism	1
ASV168	Phenylalanine metabolism	1
ASV222	Phenylalanine metabolism	1
ASV27	Phenylalanine metabolism	1
ASV340	Phenylalanine metabolism	1
ASV369	Phenylalanine metabolism	1
ASV383	Phenylalanine metabolism	1
ASV389	Phenylalanine metabolism	1
ASV416	Phenylalanine metabolism	1
ASV418	Phenylalanine metabolism	1
ASV46	Phenylalanine metabolism	1
ASV491	Phenylalanine metabolism	1
ASV575	Phenylalanine metabolism	1
ASV599	Phenylalanine metabolism	1
ASV671	Phenylalanine metabolism	1
ASV681	Phenylalanine metabolism	1
ASV709	Phenylalanine metabolism	1
ASV743	Phenylalanine metabolism	1

ASV875	Phenylalanine metabolism	1
ASV994	Phenylalanine metabolism	1
ASV199	Phenylalanine metabolism	1
ASV1026	Benzoate degradation	1
ASV137	Benzoate degradation	1
ASV168	Benzoate degradation	1
ASV222	Benzoate degradation	1
ASV27	Benzoate degradation	1
ASV340	Benzoate degradation	1
ASV369	Benzoate degradation	1
ASV383	Benzoate degradation	1
ASV389	Benzoate degradation	1
ASV416	Benzoate degradation	1
ASV418	Benzoate degradation	1
ASV46	Benzoate degradation	1
ASV491	Benzoate degradation	1
ASV575	Benzoate degradation	1
ASV599	Benzoate degradation	1
ASV671	Benzoate degradation	1
ASV681	Benzoate degradation	1
ASV709	Benzoate degradation	1
ASV743	Benzoate degradation	1
ASV875	Benzoate degradation	1
ASV994	Benzoate degradation	1
ASV199	Benzoate degradation	1
ASV1026	Caprolactam degradation	1
ASV137	Caprolactam degradation	1
ASV168	Caprolactam degradation	1
ASV222	Caprolactam degradation	1
ASV27	Caprolactam degradation	1
ASV340	Caprolactam degradation	1
ASV369	Caprolactam degradation	1
ASV383	Caprolactam degradation	1
ASV389	Caprolactam degradation	1
ASV416	Caprolactam degradation	1
ASV418	Caprolactam degradation	1
ASV46	Caprolactam degradation	1
ASV491	Caprolactam degradation	1
ASV575	Caprolactam degradation	1
ASV599	Caprolactam degradation	1
ASV671	Caprolactam degradation	1
ASV681	Caprolactam degradation	1
ASV709	Caprolactam degradation	1
ASV743	Caprolactam degradation	1

ASV875	Caprolactam degradation	1
ASV994	Caprolactam degradation	1
ASV199	Caprolactam degradation	1
ASV1026	Carbon fixation pathways in prokaryotes	1
ASV137	Carbon fixation pathways in prokaryotes	1
ASV168	Carbon fixation pathways in prokaryotes	1
ASV222	Carbon fixation pathways in prokaryotes	1
ASV27	Carbon fixation pathways in prokaryotes	1
ASV340	Carbon fixation pathways in prokaryotes	1
ASV369	Carbon fixation pathways in prokaryotes	1
ASV383	Carbon fixation pathways in prokaryotes	1
ASV389	Carbon fixation pathways in prokaryotes	1
ASV416	Carbon fixation pathways in prokaryotes	1
ASV418	Carbon fixation pathways in prokaryotes	1
ASV46	Carbon fixation pathways in prokaryotes	1
ASV491	Carbon fixation pathways in prokaryotes	1
ASV575	Carbon fixation pathways in prokaryotes	1
ASV599	Carbon fixation pathways in prokaryotes	1
ASV671	Carbon fixation pathways in prokaryotes	1
ASV681	Carbon fixation pathways in prokaryotes	1
ASV709	Carbon fixation pathways in prokaryotes	1
ASV743	Carbon fixation pathways in prokaryotes	1
ASV875	Carbon fixation pathways in prokaryotes	1
ASV994	Carbon fixation pathways in prokaryotes	1
ASV199	Carbon fixation pathways in prokaryotes	1
ASV1026	Glycolysis / Gluconeogenesis	-1
ASV137	Glycolysis / Gluconeogenesis	-1
ASV168	Glycolysis / Gluconeogenesis	-1
ASV222	Glycolysis / Gluconeogenesis	-1
ASV27	Glycolysis / Gluconeogenesis	-1
ASV340	Glycolysis / Gluconeogenesis	-1
ASV369	Glycolysis / Gluconeogenesis	-1
ASV383	Glycolysis / Gluconeogenesis	-1
ASV389	Glycolysis / Gluconeogenesis	-1
ASV416	Glycolysis / Gluconeogenesis	-1
ASV418	Glycolysis / Gluconeogenesis	-1
ASV46	Glycolysis / Gluconeogenesis	-1
ASV491	Glycolysis / Gluconeogenesis	-1
ASV575	Glycolysis / Gluconeogenesis	-1
ASV599	Glycolysis / Gluconeogenesis	-1
ASV671	Glycolysis / Gluconeogenesis	-1
ASV681	Glycolysis / Gluconeogenesis	-1
ASV709	Glycolysis / Gluconeogenesis	-1
ASV743	Glycolysis / Gluconeogenesis	-1

ASV875	Glycolysis / Gluconeogenesis	-1
ASV994	Glycolysis / Gluconeogenesis	-1
ASV199	Glycolysis / Gluconeogenesis	-1
ASV1026	Terpenoid backbone biosynthesis	1
ASV137	Terpenoid backbone biosynthesis	1
ASV168	Terpenoid backbone biosynthesis	1
ASV222	Terpenoid backbone biosynthesis	1
ASV27	Terpenoid backbone biosynthesis	1
ASV340	Terpenoid backbone biosynthesis	1
ASV369	Terpenoid backbone biosynthesis	1
ASV383	Terpenoid backbone biosynthesis	1
ASV389	Terpenoid backbone biosynthesis	1
ASV416	Terpenoid backbone biosynthesis	1
ASV418	Terpenoid backbone biosynthesis	1
ASV46	Terpenoid backbone biosynthesis	1
ASV491	Terpenoid backbone biosynthesis	1
ASV575	Terpenoid backbone biosynthesis	1
ASV599	Terpenoid backbone biosynthesis	1
ASV671	Terpenoid backbone biosynthesis	1
ASV681	Terpenoid backbone biosynthesis	1
ASV709	Terpenoid backbone biosynthesis	1
ASV743	Terpenoid backbone biosynthesis	1
ASV875	Terpenoid backbone biosynthesis	1
ASV994	Terpenoid backbone biosynthesis	1
ASV199	Terpenoid backbone biosynthesis	1
ASV1026	Ubiquinone and other terpenoid-quinone biosynthesis	1
ASV137	Ubiquinone and other terpenoid-quinone biosynthesis	1
ASV168	Ubiquinone and other terpenoid-quinone biosynthesis	1
ASV222	Ubiquinone and other terpenoid-quinone biosynthesis	1
ASV27	Ubiquinone and other terpenoid-quinone biosynthesis	1
ASV340	Ubiquinone and other terpenoid-quinone biosynthesis	1
ASV369	Ubiquinone and other terpenoid-quinone biosynthesis	1
ASV383	Ubiquinone and other terpenoid-quinone biosynthesis	1
ASV389	Ubiquinone and other terpenoid-quinone biosynthesis	1
ASV416	Ubiquinone and other terpenoid-quinone biosynthesis	1
ASV418	Ubiquinone and other terpenoid-quinone biosynthesis	1
ASV46	Ubiquinone and other terpenoid-quinone biosynthesis	1
ASV491	Ubiquinone and other terpenoid-quinone biosynthesis	1
ASV575	Ubiquinone and other terpenoid-quinone biosynthesis	1
ASV599	Ubiquinone and other terpenoid-quinone biosynthesis	1
ASV671	Ubiquinone and other terpenoid-quinone biosynthesis	1
ASV681	Ubiquinone and other terpenoid-quinone biosynthesis	1
ASV709	Ubiquinone and other terpenoid-quinone biosynthesis	1
ASV743	Ubiquinone and other terpenoid-quinone biosynthesis	1

ASV875	Ubiquinone and other terpenoid-quinone biosynthesis	1
ASV994	Ubiquinone and other terpenoid-quinone biosynthesis	1
ASV199	Ubiquinone and other terpenoid-quinone biosynthesis	1
ASV1026	Aminobenzoate degradation	1
ASV137	Aminobenzoate degradation	1
ASV168	Aminobenzoate degradation	1
ASV222	Aminobenzoate degradation	1
ASV27	Aminobenzoate degradation	1
ASV340	Aminobenzoate degradation	1
ASV369	Aminobenzoate degradation	1
ASV383	Aminobenzoate degradation	1
ASV389	Aminobenzoate degradation	1
ASV416	Aminobenzoate degradation	1
ASV418	Aminobenzoate degradation	1
ASV46	Aminobenzoate degradation	1
ASV491	Aminobenzoate degradation	1
ASV575	Aminobenzoate degradation	1
ASV599	Aminobenzoate degradation	1
ASV671	Aminobenzoate degradation	1
ASV681	Aminobenzoate degradation	1
ASV709	Aminobenzoate degradation	1
ASV743	Aminobenzoate degradation	1
ASV875	Aminobenzoate degradation	1
ASV994	Aminobenzoate degradation	1
ASV199	Aminobenzoate degradation	1
ASV1026	Chlorocyclohexane and chlorobenzene degradation	1
ASV137	Chlorocyclohexane and chlorobenzene degradation	1
ASV168	Chlorocyclohexane and chlorobenzene degradation	1
ASV222	Chlorocyclohexane and chlorobenzene degradation	1
ASV27	Chlorocyclohexane and chlorobenzene degradation	1
ASV340	Chlorocyclohexane and chlorobenzene degradation	1
ASV369	Chlorocyclohexane and chlorobenzene degradation	1
ASV383	Chlorocyclohexane and chlorobenzene degradation	1
ASV389	Chlorocyclohexane and chlorobenzene degradation	1
ASV416	Chlorocyclohexane and chlorobenzene degradation	1
ASV418	Chlorocyclohexane and chlorobenzene degradation	1
ASV46	Chlorocyclohexane and chlorobenzene degradation	1
ASV491	Chlorocyclohexane and chlorobenzene degradation	1
ASV575	Chlorocyclohexane and chlorobenzene degradation	1
ASV599	Chlorocyclohexane and chlorobenzene degradation	1
ASV671	Chlorocyclohexane and chlorobenzene degradation	1
ASV681	Chlorocyclohexane and chlorobenzene degradation	1
ASV709	Chlorocyclohexane and chlorobenzene degradation	1
ASV743	Chlorocyclohexane and chlorobenzene degradation	1

ASV875	Chlorocyclohexane and chlorobenzene degradation	1
ASV994	Chlorocyclohexane and chlorobenzene degradation	1
ASV199	Chlorocyclohexane and chlorobenzene degradation	1
ASV1026	Fluorobenzoate degradation	1
ASV137	Fluorobenzoate degradation	1
ASV168	Fluorobenzoate degradation	1
ASV222	Fluorobenzoate degradation	1
ASV27	Fluorobenzoate degradation	1
ASV340	Fluorobenzoate degradation	1
ASV369	Fluorobenzoate degradation	1
ASV383	Fluorobenzoate degradation	1
ASV389	Fluorobenzoate degradation	1
ASV416	Fluorobenzoate degradation	1
ASV418	Fluorobenzoate degradation	1
ASV46	Fluorobenzoate degradation	1
ASV491	Fluorobenzoate degradation	1
ASV575	Fluorobenzoate degradation	1
ASV599	Fluorobenzoate degradation	1
ASV671	Fluorobenzoate degradation	1
ASV681	Fluorobenzoate degradation	1
ASV709	Fluorobenzoate degradation	1
ASV743	Fluorobenzoate degradation	1
ASV875	Fluorobenzoate degradation	1
ASV994	Fluorobenzoate degradation	1
ASV199	Fluorobenzoate degradation	1
ASV1026	Carbon fixation in photosynthetic organisms	-1
ASV137	Carbon fixation in photosynthetic organisms	-1
ASV168	Carbon fixation in photosynthetic organisms	-1
ASV222	Carbon fixation in photosynthetic organisms	-1
ASV27	Carbon fixation in photosynthetic organisms	-1
ASV340	Carbon fixation in photosynthetic organisms	-1
ASV369	Carbon fixation in photosynthetic organisms	-1
ASV383	Carbon fixation in photosynthetic organisms	-1
ASV389	Carbon fixation in photosynthetic organisms	-1
ASV416	Carbon fixation in photosynthetic organisms	-1
ASV418	Carbon fixation in photosynthetic organisms	-1
ASV46	Carbon fixation in photosynthetic organisms	-1
ASV491	Carbon fixation in photosynthetic organisms	-1
ASV575	Carbon fixation in photosynthetic organisms	-1
ASV599	Carbon fixation in photosynthetic organisms	-1
ASV671	Carbon fixation in photosynthetic organisms	-1
ASV681	Carbon fixation in photosynthetic organisms	-1
ASV709	Carbon fixation in photosynthetic organisms	-1
ASV743	Carbon fixation in photosynthetic organisms	-1

ASV875	Carbon fixation in photosynthetic organisms	-1
ASV994	Carbon fixation in photosynthetic organisms	-1
ASV199	Carbon fixation in photosynthetic organisms	-1
ASV1026	Valine, leucine and isoleucine degradation	1
ASV137	Valine, leucine and isoleucine degradation	1
ASV168	Valine, leucine and isoleucine degradation	1
ASV222	Valine, leucine and isoleucine degradation	1
ASV27	Valine, leucine and isoleucine degradation	1
ASV340	Valine, leucine and isoleucine degradation	1
ASV369	Valine, leucine and isoleucine degradation	1
ASV383	Valine, leucine and isoleucine degradation	1
ASV389	Valine, leucine and isoleucine degradation	1
ASV416	Valine, leucine and isoleucine degradation	1
ASV418	Valine, leucine and isoleucine degradation	1
ASV46	Valine, leucine and isoleucine degradation	1
ASV491	Valine, leucine and isoleucine degradation	1
ASV575	Valine, leucine and isoleucine degradation	1
ASV599	Valine, leucine and isoleucine degradation	1
ASV671	Valine, leucine and isoleucine degradation	1
ASV681	Valine, leucine and isoleucine degradation	1
ASV709	Valine, leucine and isoleucine degradation	1
ASV743	Valine, leucine and isoleucine degradation	1
ASV875	Valine, leucine and isoleucine degradation	1
ASV994	Valine, leucine and isoleucine degradation	1
ASV199	Valine, leucine and isoleucine degradation	1
ASV1026	Geraniol degradation	1
ASV137	Geraniol degradation	1
ASV168	Geraniol degradation	1
ASV222	Geraniol degradation	1
ASV27	Geraniol degradation	1
ASV340	Geraniol degradation	1
ASV369	Geraniol degradation	1
ASV383	Geraniol degradation	1
ASV389	Geraniol degradation	1
ASV416	Geraniol degradation	1
ASV418	Geraniol degradation	1
ASV46	Geraniol degradation	1
ASV491	Geraniol degradation	1
ASV575	Geraniol degradation	1
ASV599	Geraniol degradation	1
ASV671	Geraniol degradation	1
ASV681	Geraniol degradation	1
ASV709	Geraniol degradation	1
ASV743	Geraniol degradation	1

ASV875	Geraniol degradation	1
ASV994	Geraniol degradation	1
ASV199	Geraniol degradation	1
ASV1026	Amino sugar and nucleotide sugar metabolism	-1
ASV137	Amino sugar and nucleotide sugar metabolism	-1
ASV168	Amino sugar and nucleotide sugar metabolism	-1
ASV222	Amino sugar and nucleotide sugar metabolism	-1
ASV27	Amino sugar and nucleotide sugar metabolism	-1
ASV340	Amino sugar and nucleotide sugar metabolism	-1
ASV369	Amino sugar and nucleotide sugar metabolism	-1
ASV383	Amino sugar and nucleotide sugar metabolism	-1
ASV389	Amino sugar and nucleotide sugar metabolism	-1
ASV416	Amino sugar and nucleotide sugar metabolism	-1
ASV418	Amino sugar and nucleotide sugar metabolism	-1
ASV46	Amino sugar and nucleotide sugar metabolism	-1
ASV491	Amino sugar and nucleotide sugar metabolism	-1
ASV575	Amino sugar and nucleotide sugar metabolism	-1
ASV599	Amino sugar and nucleotide sugar metabolism	-1
ASV671	Amino sugar and nucleotide sugar metabolism	-1
ASV681	Amino sugar and nucleotide sugar metabolism	-1
ASV709	Amino sugar and nucleotide sugar metabolism	-1
ASV743	Amino sugar and nucleotide sugar metabolism	-1
ASV875	Amino sugar and nucleotide sugar metabolism	-1
ASV994	Amino sugar and nucleotide sugar metabolism	-1
ASV199	Amino sugar and nucleotide sugar metabolism	-1
ASV1026	Atrazine degradation	-1
ASV137	Atrazine degradation	-1
ASV168	Atrazine degradation	-1
ASV222	Atrazine degradation	-1
ASV27	Atrazine degradation	-1
ASV340	Atrazine degradation	-1
ASV369	Atrazine degradation	-1
ASV383	Atrazine degradation	-1
ASV389	Atrazine degradation	-1
ASV416	Atrazine degradation	-1
ASV418	Atrazine degradation	-1
ASV46	Atrazine degradation	-1
ASV491	Atrazine degradation	-1
ASV575	Atrazine degradation	-1
ASV599	Atrazine degradation	-1
ASV671	Atrazine degradation	-1
ASV681	Atrazine degradation	-1
ASV709	Atrazine degradation	-1
ASV743	Atrazine degradation	-1

ASV875	Atrazine degradation	-1
ASV994	Atrazine degradation	-1
ASV199	Atrazine degradation	-1
ASV1026	Synthesis and degradation of ketone bodies	1
ASV137	Synthesis and degradation of ketone bodies	1
ASV168	Synthesis and degradation of ketone bodies	1
ASV222	Synthesis and degradation of ketone bodies	1
ASV27	Synthesis and degradation of ketone bodies	1
ASV340	Synthesis and degradation of ketone bodies	1
ASV369	Synthesis and degradation of ketone bodies	1
ASV383	Synthesis and degradation of ketone bodies	1
ASV389	Synthesis and degradation of ketone bodies	1
ASV416	Synthesis and degradation of ketone bodies	1
ASV418	Synthesis and degradation of ketone bodies	1
ASV46	Synthesis and degradation of ketone bodies	1
ASV491	Synthesis and degradation of ketone bodies	1
ASV575	Synthesis and degradation of ketone bodies	1
ASV599	Synthesis and degradation of ketone bodies	1
ASV671	Synthesis and degradation of ketone bodies	1
ASV681	Synthesis and degradation of ketone bodies	1
ASV709	Synthesis and degradation of ketone bodies	1
ASV743	Synthesis and degradation of ketone bodies	1
ASV875	Synthesis and degradation of ketone bodies	1
ASV994	Synthesis and degradation of ketone bodies	1
ASV199	Synthesis and degradation of ketone bodies	1

Table S6 Correlation analysis between different strains and metabolic function（EQ&MH&Pathway）

EQ&MH&Pathway		
Var1	Var2	cor
ASV137	Chloroalkane and chloroalkene degradation	1
ASV168	Chloroalkane and chloroalkene degradation	1
ASV27	Chloroalkane and chloroalkene degradation	1
ASV386	Chloroalkane and chloroalkene degradation	1
ASV389	Chloroalkane and chloroalkene degradation	1
ASV416	Chloroalkane and chloroalkene degradation	1
ASV46	Chloroalkane and chloroalkene degradation	1
ASV540	Chloroalkane and chloroalkene degradation	1
ASV575	Chloroalkane and chloroalkene degradation	1
ASV599	Chloroalkane and chloroalkene degradation	1
ASV647	Chloroalkane and chloroalkene degradation	1
ASV671	Chloroalkane and chloroalkene degradation	1
ASV709	Chloroalkane and chloroalkene degradation	1
ASV743	Chloroalkane and chloroalkene degradation	1
ASV994	Chloroalkane and chloroalkene degradation	1
ASV137	Phenylalanine metabolism	-1
ASV168	Phenylalanine metabolism	-1
ASV27	Phenylalanine metabolism	-1
ASV386	Phenylalanine metabolism	-1
ASV389	Phenylalanine metabolism	-1
ASV416	Phenylalanine metabolism	-1
ASV46	Phenylalanine metabolism	-1
ASV540	Phenylalanine metabolism	-1
ASV575	Phenylalanine metabolism	-1
ASV599	Phenylalanine metabolism	-1
ASV647	Phenylalanine metabolism	-1
ASV671	Phenylalanine metabolism	-1
ASV709	Phenylalanine metabolism	-1
ASV743	Phenylalanine metabolism	-1
ASV994	Phenylalanine metabolism	-1
ASV137	Benzoate degradation	-1
ASV168	Benzoate degradation	-1
ASV27	Benzoate degradation	-1
ASV386	Benzoate degradation	-1
ASV389	Benzoate degradation	-1
ASV416	Benzoate degradation	-1
ASV46	Benzoate degradation	-1
ASV540	Benzoate degradation	-1
ASV575	Benzoate degradation	-1
ASV599	Benzoate degradation	-1
ASV647	Benzoate degradation	-1

ASV671	Benzoate degradation	-1
ASV709	Benzoate degradation	-1
ASV743	Benzoate degradation	-1
ASV994	Benzoate degradation	-1
ASV137	Caprolactam degradation	-1
ASV168	Caprolactam degradation	-1
ASV27	Caprolactam degradation	-1
ASV386	Caprolactam degradation	-1
ASV389	Caprolactam degradation	-1
ASV416	Caprolactam degradation	-1
ASV46	Caprolactam degradation	-1
ASV540	Caprolactam degradation	-1
ASV575	Caprolactam degradation	-1
ASV599	Caprolactam degradation	-1
ASV647	Caprolactam degradation	-1
ASV671	Caprolactam degradation	-1
ASV709	Caprolactam degradation	-1
ASV743	Caprolactam degradation	-1
ASV994	Caprolactam degradation	-1
ASV137	Carbon fixation pathways in prokaryotes	-1
ASV168	Carbon fixation pathways in prokaryotes	-1
ASV27	Carbon fixation pathways in prokaryotes	-1
ASV386	Carbon fixation pathways in prokaryotes	-1
ASV389	Carbon fixation pathways in prokaryotes	-1
ASV416	Carbon fixation pathways in prokaryotes	-1
ASV46	Carbon fixation pathways in prokaryotes	-1
ASV540	Carbon fixation pathways in prokaryotes	-1
ASV575	Carbon fixation pathways in prokaryotes	-1
ASV599	Carbon fixation pathways in prokaryotes	-1
ASV647	Carbon fixation pathways in prokaryotes	-1
ASV671	Carbon fixation pathways in prokaryotes	-1
ASV709	Carbon fixation pathways in prokaryotes	-1
ASV743	Carbon fixation pathways in prokaryotes	-1
ASV994	Carbon fixation pathways in prokaryotes	-1
ASV137	Glycolysis / Gluconeogenesis	1
ASV168	Glycolysis / Gluconeogenesis	1
ASV27	Glycolysis / Gluconeogenesis	1
ASV386	Glycolysis / Gluconeogenesis	1
ASV389	Glycolysis / Gluconeogenesis	1
ASV416	Glycolysis / Gluconeogenesis	1
ASV46	Glycolysis / Gluconeogenesis	1
ASV540	Glycolysis / Gluconeogenesis	1
ASV575	Glycolysis / Gluconeogenesis	1
ASV599	Glycolysis / Gluconeogenesis	1

ASV647	Glycolysis / Gluconeogenesis	1
ASV671	Glycolysis / Gluconeogenesis	1
ASV709	Glycolysis / Gluconeogenesis	1
ASV743	Glycolysis / Gluconeogenesis	1
ASV994	Glycolysis / Gluconeogenesis	1
ASV137	Terpenoid backbone biosynthesis	-1
ASV168	Terpenoid backbone biosynthesis	-1
ASV27	Terpenoid backbone biosynthesis	-1
ASV386	Terpenoid backbone biosynthesis	-1
ASV389	Terpenoid backbone biosynthesis	-1
ASV416	Terpenoid backbone biosynthesis	-1
ASV46	Terpenoid backbone biosynthesis	-1
ASV540	Terpenoid backbone biosynthesis	-1
ASV575	Terpenoid backbone biosynthesis	-1
ASV599	Terpenoid backbone biosynthesis	-1
ASV647	Terpenoid backbone biosynthesis	-1
ASV671	Terpenoid backbone biosynthesis	-1
ASV709	Terpenoid backbone biosynthesis	-1
ASV743	Terpenoid backbone biosynthesis	-1
ASV994	Terpenoid backbone biosynthesis	-1
ASV137	Ubiquinone and other terpenoid-quinone biosynthesis	-1
ASV168	Ubiquinone and other terpenoid-quinone biosynthesis	-1
ASV27	Ubiquinone and other terpenoid-quinone biosynthesis	-1
ASV386	Ubiquinone and other terpenoid-quinone biosynthesis	-1
ASV389	Ubiquinone and other terpenoid-quinone biosynthesis	-1
ASV416	Ubiquinone and other terpenoid-quinone biosynthesis	-1
ASV46	Ubiquinone and other terpenoid-quinone biosynthesis	-1
ASV540	Ubiquinone and other terpenoid-quinone biosynthesis	-1
ASV575	Ubiquinone and other terpenoid-quinone biosynthesis	-1
ASV599	Ubiquinone and other terpenoid-quinone biosynthesis	-1
ASV647	Ubiquinone and other terpenoid-quinone biosynthesis	-1
ASV671	Ubiquinone and other terpenoid-quinone biosynthesis	-1
ASV709	Ubiquinone and other terpenoid-quinone biosynthesis	-1
ASV743	Ubiquinone and other terpenoid-quinone biosynthesis	-1
ASV994	Ubiquinone and other terpenoid-quinone biosynthesis	-1
ASV137	Aminobenzoate degradation	-1
ASV168	Aminobenzoate degradation	-1
ASV27	Aminobenzoate degradation	-1
ASV386	Aminobenzoate degradation	-1
ASV389	Aminobenzoate degradation	-1
ASV416	Aminobenzoate degradation	-1
ASV46	Aminobenzoate degradation	-1
ASV540	Aminobenzoate degradation	-1
ASV575	Aminobenzoate degradation	-1

ASV599	Aminobenzoate degradation	-1
ASV647	Aminobenzoate degradation	-1
ASV671	Aminobenzoate degradation	-1
ASV709	Aminobenzoate degradation	-1
ASV743	Aminobenzoate degradation	-1
ASV994	Aminobenzoate degradation	-1
ASV137	Chlorocyclohexane and chlorobenzene degradation	-1
ASV168	Chlorocyclohexane and chlorobenzene degradation	-1
ASV27	Chlorocyclohexane and chlorobenzene degradation	-1
ASV386	Chlorocyclohexane and chlorobenzene degradation	-1
ASV389	Chlorocyclohexane and chlorobenzene degradation	-1
ASV416	Chlorocyclohexane and chlorobenzene degradation	-1
ASV46	Chlorocyclohexane and chlorobenzene degradation	-1
ASV540	Chlorocyclohexane and chlorobenzene degradation	-1
ASV575	Chlorocyclohexane and chlorobenzene degradation	-1
ASV599	Chlorocyclohexane and chlorobenzene degradation	-1
ASV647	Chlorocyclohexane and chlorobenzene degradation	-1
ASV671	Chlorocyclohexane and chlorobenzene degradation	-1
ASV709	Chlorocyclohexane and chlorobenzene degradation	-1
ASV743	Chlorocyclohexane and chlorobenzene degradation	-1
ASV994	Chlorocyclohexane and chlorobenzene degradation	-1
ASV137	Fluorobenzoate degradation	-1
ASV168	Fluorobenzoate degradation	-1
ASV27	Fluorobenzoate degradation	-1
ASV386	Fluorobenzoate degradation	-1
ASV389	Fluorobenzoate degradation	-1
ASV416	Fluorobenzoate degradation	-1
ASV46	Fluorobenzoate degradation	-1
ASV540	Fluorobenzoate degradation	-1
ASV575	Fluorobenzoate degradation	-1
ASV599	Fluorobenzoate degradation	-1
ASV647	Fluorobenzoate degradation	-1
ASV671	Fluorobenzoate degradation	-1
ASV709	Fluorobenzoate degradation	-1
ASV743	Fluorobenzoate degradation	-1
ASV994	Fluorobenzoate degradation	-1
ASV137	Carbon fixation in photosynthetic organisms	1
ASV168	Carbon fixation in photosynthetic organisms	1
ASV27	Carbon fixation in photosynthetic organisms	1
ASV386	Carbon fixation in photosynthetic organisms	1
ASV389	Carbon fixation in photosynthetic organisms	1
ASV416	Carbon fixation in photosynthetic organisms	1
ASV46	Carbon fixation in photosynthetic organisms	1
ASV540	Carbon fixation in photosynthetic organisms	1

ASV575	Carbon fixation in photosynthetic organisms	1
ASV599	Carbon fixation in photosynthetic organisms	1
ASV647	Carbon fixation in photosynthetic organisms	1
ASV671	Carbon fixation in photosynthetic organisms	1
ASV709	Carbon fixation in photosynthetic organisms	1
ASV743	Carbon fixation in photosynthetic organisms	1
ASV994	Carbon fixation in photosynthetic organisms	1
ASV137	Valine, leucine and isoleucine degradation	-1
ASV168	Valine, leucine and isoleucine degradation	-1
ASV27	Valine, leucine and isoleucine degradation	-1
ASV386	Valine, leucine and isoleucine degradation	-1
ASV389	Valine, leucine and isoleucine degradation	-1
ASV416	Valine, leucine and isoleucine degradation	-1
ASV46	Valine, leucine and isoleucine degradation	-1
ASV540	Valine, leucine and isoleucine degradation	-1
ASV575	Valine, leucine and isoleucine degradation	-1
ASV599	Valine, leucine and isoleucine degradation	-1
ASV647	Valine, leucine and isoleucine degradation	-1
ASV671	Valine, leucine and isoleucine degradation	-1
ASV709	Valine, leucine and isoleucine degradation	-1
ASV743	Valine, leucine and isoleucine degradation	-1
ASV994	Valine, leucine and isoleucine degradation	-1
ASV137	Geraniol degradation	-1
ASV168	Geraniol degradation	-1
ASV27	Geraniol degradation	-1
ASV386	Geraniol degradation	-1
ASV389	Geraniol degradation	-1
ASV416	Geraniol degradation	-1
ASV46	Geraniol degradation	-1
ASV540	Geraniol degradation	-1
ASV575	Geraniol degradation	-1
ASV599	Geraniol degradation	-1
ASV647	Geraniol degradation	-1
ASV671	Geraniol degradation	-1
ASV709	Geraniol degradation	-1
ASV743	Geraniol degradation	-1
ASV994	Geraniol degradation	-1
ASV137	Amino sugar and nucleotide sugar metabolism	1
ASV168	Amino sugar and nucleotide sugar metabolism	1
ASV27	Amino sugar and nucleotide sugar metabolism	1
ASV386	Amino sugar and nucleotide sugar metabolism	1
ASV389	Amino sugar and nucleotide sugar metabolism	1
ASV416	Amino sugar and nucleotide sugar metabolism	1
ASV46	Amino sugar and nucleotide sugar metabolism	1

ASV540	Amino sugar and nucleotide sugar metabolism	1
ASV575	Amino sugar and nucleotide sugar metabolism	1
ASV599	Amino sugar and nucleotide sugar metabolism	1
ASV647	Amino sugar and nucleotide sugar metabolism	1
ASV671	Amino sugar and nucleotide sugar metabolism	1
ASV709	Amino sugar and nucleotide sugar metabolism	1
ASV743	Amino sugar and nucleotide sugar metabolism	1
ASV994	Amino sugar and nucleotide sugar metabolism	1
ASV137	Atrazine degradation	-1
ASV168	Atrazine degradation	-1
ASV27	Atrazine degradation	-1
ASV386	Atrazine degradation	-1
ASV389	Atrazine degradation	-1
ASV416	Atrazine degradation	-1
ASV46	Atrazine degradation	-1
ASV540	Atrazine degradation	-1
ASV575	Atrazine degradation	-1
ASV599	Atrazine degradation	-1
ASV647	Atrazine degradation	-1
ASV671	Atrazine degradation	-1
ASV709	Atrazine degradation	-1
ASV743	Atrazine degradation	-1
ASV994	Atrazine degradation	-1
ASV137	Synthesis and degradation of ketone bodies	-1
ASV168	Synthesis and degradation of ketone bodies	-1
ASV27	Synthesis and degradation of ketone bodies	-1
ASV386	Synthesis and degradation of ketone bodies	-1
ASV389	Synthesis and degradation of ketone bodies	-1
ASV416	Synthesis and degradation of ketone bodies	-1
ASV46	Synthesis and degradation of ketone bodies	-1
ASV540	Synthesis and degradation of ketone bodies	-1
ASV575	Synthesis and degradation of ketone bodies	-1
ASV599	Synthesis and degradation of ketone bodies	-1
ASV647	Synthesis and degradation of ketone bodies	-1
ASV671	Synthesis and degradation of ketone bodies	-1
ASV709	Synthesis and degradation of ketone bodies	-1
ASV743	Synthesis and degradation of ketone bodies	-1
ASV994	Synthesis and degradation of ketone bodies	-1

Table S7 Correlation analysis between different strains and metabolic function（BN&MH&Pathway）

BN&MH&Pathway		
Var1	Var2	cor
ASV1089	Chloroalkane and chloroalkene degradation	-1
ASV137	Chloroalkane and chloroalkene degradation	1
ASV168	Chloroalkane and chloroalkene degradation	1
ASV27	Chloroalkane and chloroalkene degradation	1
ASV386	Chloroalkane and chloroalkene degradation	1
ASV389	Chloroalkane and chloroalkene degradation	1
ASV416	Chloroalkane and chloroalkene degradation	1
ASV46	Chloroalkane and chloroalkene degradation	1
ASV540	Chloroalkane and chloroalkene degradation	1
ASV575	Chloroalkane and chloroalkene degradation	1
ASV599	Chloroalkane and chloroalkene degradation	1
ASV647	Chloroalkane and chloroalkene degradation	1
ASV66	Chloroalkane and chloroalkene degradation	-1
ASV671	Chloroalkane and chloroalkene degradation	1
ASV709	Chloroalkane and chloroalkene degradation	1
ASV743	Chloroalkane and chloroalkene degradation	1
ASV994	Chloroalkane and chloroalkene degradation	1
ASV1089	Phenylalanine metabolism	-1
ASV137	Phenylalanine metabolism	1
ASV168	Phenylalanine metabolism	1
ASV27	Phenylalanine metabolism	1
ASV386	Phenylalanine metabolism	1
ASV389	Phenylalanine metabolism	1
ASV416	Phenylalanine metabolism	1
ASV46	Phenylalanine metabolism	1
ASV540	Phenylalanine metabolism	1
ASV575	Phenylalanine metabolism	1
ASV599	Phenylalanine metabolism	1
ASV647	Phenylalanine metabolism	1
ASV66	Phenylalanine metabolism	-1
ASV671	Phenylalanine metabolism	1
ASV709	Phenylalanine metabolism	1
ASV743	Phenylalanine metabolism	1
ASV994	Phenylalanine metabolism	1
ASV1089	Benzoate degradation	-1
ASV137	Benzoate degradation	1
ASV168	Benzoate degradation	1
ASV27	Benzoate degradation	1
ASV386	Benzoate degradation	1
ASV389	Benzoate degradation	1
ASV416	Benzoate degradation	1

ASV46	Benzoate degradation	1
ASV540	Benzoate degradation	1
ASV575	Benzoate degradation	1
ASV599	Benzoate degradation	1
ASV647	Benzoate degradation	1
ASV66	Benzoate degradation	-1
ASV671	Benzoate degradation	1
ASV709	Benzoate degradation	1
ASV743	Benzoate degradation	1
ASV994	Benzoate degradation	1
ASV1089	Caprolactam degradation	-1
ASV137	Caprolactam degradation	1
ASV168	Caprolactam degradation	1
ASV27	Caprolactam degradation	1
ASV386	Caprolactam degradation	1
ASV389	Caprolactam degradation	1
ASV416	Caprolactam degradation	1
ASV46	Caprolactam degradation	1
ASV540	Caprolactam degradation	1
ASV575	Caprolactam degradation	1
ASV599	Caprolactam degradation	1
ASV647	Caprolactam degradation	1
ASV66	Caprolactam degradation	-1
ASV671	Caprolactam degradation	1
ASV709	Caprolactam degradation	1
ASV743	Caprolactam degradation	1
ASV994	Caprolactam degradation	1
ASV1089	Carbon fixation pathways in prokaryotes	-1
ASV137	Carbon fixation pathways in prokaryotes	1
ASV168	Carbon fixation pathways in prokaryotes	1
ASV27	Carbon fixation pathways in prokaryotes	1
ASV386	Carbon fixation pathways in prokaryotes	1
ASV389	Carbon fixation pathways in prokaryotes	1
ASV416	Carbon fixation pathways in prokaryotes	1
ASV46	Carbon fixation pathways in prokaryotes	1
ASV540	Carbon fixation pathways in prokaryotes	1
ASV575	Carbon fixation pathways in prokaryotes	1
ASV599	Carbon fixation pathways in prokaryotes	1
ASV647	Carbon fixation pathways in prokaryotes	1
ASV66	Carbon fixation pathways in prokaryotes	-1
ASV671	Carbon fixation pathways in prokaryotes	1
ASV709	Carbon fixation pathways in prokaryotes	1
ASV743	Carbon fixation pathways in prokaryotes	1
ASV994	Carbon fixation pathways in prokaryotes	1

ASV1089	Glycolysis / Gluconeogenesis	1
ASV137	Glycolysis / Gluconeogenesis	-1
ASV168	Glycolysis / Gluconeogenesis	-1
ASV27	Glycolysis / Gluconeogenesis	-1
ASV386	Glycolysis / Gluconeogenesis	-1
ASV389	Glycolysis / Gluconeogenesis	-1
ASV416	Glycolysis / Gluconeogenesis	-1
ASV46	Glycolysis / Gluconeogenesis	-1
ASV540	Glycolysis / Gluconeogenesis	-1
ASV575	Glycolysis / Gluconeogenesis	-1
ASV599	Glycolysis / Gluconeogenesis	-1
ASV647	Glycolysis / Gluconeogenesis	-1
ASV66	Glycolysis / Gluconeogenesis	1
ASV671	Glycolysis / Gluconeogenesis	-1
ASV709	Glycolysis / Gluconeogenesis	-1
ASV743	Glycolysis / Gluconeogenesis	-1
ASV994	Glycolysis / Gluconeogenesis	-1
ASV1089	Terpenoid backbone biosynthesis	1
ASV137	Terpenoid backbone biosynthesis	-1
ASV168	Terpenoid backbone biosynthesis	-1
ASV27	Terpenoid backbone biosynthesis	-1
ASV386	Terpenoid backbone biosynthesis	-1
ASV389	Terpenoid backbone biosynthesis	-1
ASV416	Terpenoid backbone biosynthesis	-1
ASV46	Terpenoid backbone biosynthesis	-1
ASV540	Terpenoid backbone biosynthesis	-1
ASV575	Terpenoid backbone biosynthesis	-1
ASV599	Terpenoid backbone biosynthesis	-1
ASV647	Terpenoid backbone biosynthesis	-1
ASV66	Terpenoid backbone biosynthesis	1
ASV671	Terpenoid backbone biosynthesis	-1
ASV709	Terpenoid backbone biosynthesis	-1
ASV743	Terpenoid backbone biosynthesis	-1
ASV994	Terpenoid backbone biosynthesis	-1
ASV1089	Ubiquinone and other terpenoid-quinone biosynthesis	1
ASV137	Ubiquinone and other terpenoid-quinone biosynthesis	-1
ASV168	Ubiquinone and other terpenoid-quinone biosynthesis	-1
ASV27	Ubiquinone and other terpenoid-quinone biosynthesis	-1
ASV386	Ubiquinone and other terpenoid-quinone biosynthesis	-1
ASV389	Ubiquinone and other terpenoid-quinone biosynthesis	-1
ASV416	Ubiquinone and other terpenoid-quinone biosynthesis	-1
ASV46	Ubiquinone and other terpenoid-quinone biosynthesis	-1
ASV540	Ubiquinone and other terpenoid-quinone biosynthesis	-1
ASV575	Ubiquinone and other terpenoid-quinone biosynthesis	-1

ASV599	Ubiquinone and other terpenoid-quinone biosynthesis	-1
ASV647	Ubiquinone and other terpenoid-quinone biosynthesis	-1
ASV66	Ubiquinone and other terpenoid-quinone biosynthesis	1
ASV671	Ubiquinone and other terpenoid-quinone biosynthesis	-1
ASV709	Ubiquinone and other terpenoid-quinone biosynthesis	-1
ASV743	Ubiquinone and other terpenoid-quinone biosynthesis	-1
ASV994	Ubiquinone and other terpenoid-quinone biosynthesis	-1
ASV1089	Aminobenzoate degradation	1
ASV137	Aminobenzoate degradation	-1
ASV168	Aminobenzoate degradation	-1
ASV27	Aminobenzoate degradation	-1
ASV386	Aminobenzoate degradation	-1
ASV389	Aminobenzoate degradation	-1
ASV416	Aminobenzoate degradation	-1
ASV46	Aminobenzoate degradation	-1
ASV540	Aminobenzoate degradation	-1
ASV575	Aminobenzoate degradation	-1
ASV599	Aminobenzoate degradation	-1
ASV647	Aminobenzoate degradation	-1
ASV66	Aminobenzoate degradation	1
ASV671	Aminobenzoate degradation	-1
ASV709	Aminobenzoate degradation	-1
ASV743	Aminobenzoate degradation	-1
ASV994	Aminobenzoate degradation	-1
ASV1089	Chlorocyclohexane and chlorobenzene degradation	1
ASV137	Chlorocyclohexane and chlorobenzene degradation	-1
ASV168	Chlorocyclohexane and chlorobenzene degradation	-1
ASV27	Chlorocyclohexane and chlorobenzene degradation	-1
ASV386	Chlorocyclohexane and chlorobenzene degradation	-1
ASV389	Chlorocyclohexane and chlorobenzene degradation	-1
ASV416	Chlorocyclohexane and chlorobenzene degradation	-1
ASV46	Chlorocyclohexane and chlorobenzene degradation	-1
ASV540	Chlorocyclohexane and chlorobenzene degradation	-1
ASV575	Chlorocyclohexane and chlorobenzene degradation	-1
ASV599	Chlorocyclohexane and chlorobenzene degradation	-1
ASV647	Chlorocyclohexane and chlorobenzene degradation	-1
ASV66	Chlorocyclohexane and chlorobenzene degradation	1
ASV671	Chlorocyclohexane and chlorobenzene degradation	-1
ASV709	Chlorocyclohexane and chlorobenzene degradation	-1
ASV743	Chlorocyclohexane and chlorobenzene degradation	-1
ASV994	Chlorocyclohexane and chlorobenzene degradation	-1
ASV1089	Fluorobenzoate degradation	1
ASV137	Fluorobenzoate degradation	-1
ASV168	Fluorobenzoate degradation	-1

ASV27	Fluorobenzoate degradation	-1
ASV386	Fluorobenzoate degradation	-1
ASV389	Fluorobenzoate degradation	-1
ASV416	Fluorobenzoate degradation	-1
ASV46	Fluorobenzoate degradation	-1
ASV540	Fluorobenzoate degradation	-1
ASV575	Fluorobenzoate degradation	-1
ASV599	Fluorobenzoate degradation	-1
ASV647	Fluorobenzoate degradation	-1
ASV66	Fluorobenzoate degradation	1
ASV671	Fluorobenzoate degradation	-1
ASV709	Fluorobenzoate degradation	-1
ASV743	Fluorobenzoate degradation	-1
ASV994	Fluorobenzoate degradation	-1
ASV1089	Carbon fixation in photosynthetic organisms	1
ASV137	Carbon fixation in photosynthetic organisms	-1
ASV168	Carbon fixation in photosynthetic organisms	-1
ASV27	Carbon fixation in photosynthetic organisms	-1
ASV386	Carbon fixation in photosynthetic organisms	-1
ASV389	Carbon fixation in photosynthetic organisms	-1
ASV416	Carbon fixation in photosynthetic organisms	-1
ASV46	Carbon fixation in photosynthetic organisms	-1
ASV540	Carbon fixation in photosynthetic organisms	-1
ASV575	Carbon fixation in photosynthetic organisms	-1
ASV599	Carbon fixation in photosynthetic organisms	-1
ASV647	Carbon fixation in photosynthetic organisms	-1
ASV66	Carbon fixation in photosynthetic organisms	1
ASV671	Carbon fixation in photosynthetic organisms	-1
ASV709	Carbon fixation in photosynthetic organisms	-1
ASV743	Carbon fixation in photosynthetic organisms	-1
ASV994	Carbon fixation in photosynthetic organisms	-1
ASV1089	Valine, leucine and isoleucine degradation	-1
ASV137	Valine, leucine and isoleucine degradation	1
ASV168	Valine, leucine and isoleucine degradation	1
ASV27	Valine, leucine and isoleucine degradation	1
ASV386	Valine, leucine and isoleucine degradation	1
ASV389	Valine, leucine and isoleucine degradation	1
ASV416	Valine, leucine and isoleucine degradation	1
ASV46	Valine, leucine and isoleucine degradation	1
ASV540	Valine, leucine and isoleucine degradation	1
ASV575	Valine, leucine and isoleucine degradation	1
ASV599	Valine, leucine and isoleucine degradation	1
ASV647	Valine, leucine and isoleucine degradation	1
ASV66	Valine, leucine and isoleucine degradation	-1

ASV671	Valine, leucine and isoleucine degradation	1
ASV709	Valine, leucine and isoleucine degradation	1
ASV743	Valine, leucine and isoleucine degradation	1
ASV994	Valine, leucine and isoleucine degradation	1
ASV1089	Geraniol degradation	-1
ASV137	Geraniol degradation	1
ASV168	Geraniol degradation	1
ASV27	Geraniol degradation	1
ASV386	Geraniol degradation	1
ASV389	Geraniol degradation	1
ASV416	Geraniol degradation	1
ASV46	Geraniol degradation	1
ASV540	Geraniol degradation	1
ASV575	Geraniol degradation	1
ASV599	Geraniol degradation	1
ASV647	Geraniol degradation	1
ASV66	Geraniol degradation	-1
ASV671	Geraniol degradation	1
ASV709	Geraniol degradation	1
ASV743	Geraniol degradation	1
ASV994	Geraniol degradation	1
ASV1089	Amino sugar and nucleotide sugar metabolism	1
ASV137	Amino sugar and nucleotide sugar metabolism	-1
ASV168	Amino sugar and nucleotide sugar metabolism	-1
ASV27	Amino sugar and nucleotide sugar metabolism	-1
ASV386	Amino sugar and nucleotide sugar metabolism	-1
ASV389	Amino sugar and nucleotide sugar metabolism	-1
ASV416	Amino sugar and nucleotide sugar metabolism	-1
ASV46	Amino sugar and nucleotide sugar metabolism	-1
ASV540	Amino sugar and nucleotide sugar metabolism	-1
ASV575	Amino sugar and nucleotide sugar metabolism	-1
ASV599	Amino sugar and nucleotide sugar metabolism	-1
ASV647	Amino sugar and nucleotide sugar metabolism	-1
ASV66	Amino sugar and nucleotide sugar metabolism	1
ASV671	Amino sugar and nucleotide sugar metabolism	-1
ASV709	Amino sugar and nucleotide sugar metabolism	-1
ASV743	Amino sugar and nucleotide sugar metabolism	-1
ASV994	Amino sugar and nucleotide sugar metabolism	-1
ASV1089	Atrazine degradation	-1
ASV137	Atrazine degradation	1
ASV168	Atrazine degradation	1
ASV27	Atrazine degradation	1
ASV386	Atrazine degradation	1
ASV389	Atrazine degradation	1

ASV416	Atrazine degradation	1
ASV46	Atrazine degradation	1
ASV540	Atrazine degradation	1
ASV575	Atrazine degradation	1
ASV599	Atrazine degradation	1
ASV647	Atrazine degradation	1
ASV66	Atrazine degradation	-1
ASV671	Atrazine degradation	1
ASV709	Atrazine degradation	1
ASV743	Atrazine degradation	1
ASV994	Atrazine degradation	1
ASV1089	Synthesis and degradation of ketone bodies	-1
ASV137	Synthesis and degradation of ketone bodies	1
ASV168	Synthesis and degradation of ketone bodies	1
ASV27	Synthesis and degradation of ketone bodies	1
ASV386	Synthesis and degradation of ketone bodies	1
ASV389	Synthesis and degradation of ketone bodies	1
ASV416	Synthesis and degradation of ketone bodies	1
ASV46	Synthesis and degradation of ketone bodies	1
ASV540	Synthesis and degradation of ketone bodies	1
ASV575	Synthesis and degradation of ketone bodies	1
ASV599	Synthesis and degradation of ketone bodies	1
ASV647	Synthesis and degradation of ketone bodies	1
ASV66	Synthesis and degradation of ketone bodies	-1
ASV671	Synthesis and degradation of ketone bodies	1
ASV709	Synthesis and degradation of ketone bodies	1
ASV743	Synthesis and degradation of ketone bodies	1
ASV994	Synthesis and degradation of ketone bodies	1
